# Functional Characterization of Human ProNGF and NGF Mutants: Identification of NGF P61SR100E as a “Painless” Lead Investigational Candidate for Therapeutic Applications

**DOI:** 10.1371/journal.pone.0136425

**Published:** 2015-09-15

**Authors:** Francesca Malerba, Francesca Paoletti, Bruno Bruni Ercole, Serena Materazzi, Romina Nassini, Elisabetta Coppi, Riccardo Patacchini, Simona Capsoni, Doriano Lamba, Antonino Cattaneo

**Affiliations:** 1 Neurotrophic Factors and Neurodegenerative Diseases Unit, European Brain Research Institute, “Rita Levi-Montalcini” Foundation, Rome, Italy; 2 Neurobiology Laboratory of Biology, Scuola Normale Superiore, Pisa, Italy; 3 Department of Health Sciences, Section of Clinical Pharmacology and Oncology, University of Florence, Florence, Italy; 4 Department of Pharmacology, Chiesi Farmaceutici, Parma, Italy; 5 Istituto di Cristallografia, Consiglio Nazionale delle Ricerche, Area Science Park–Basovizza, Trieste, Italy; University of Florida, UNITED STATES

## Abstract

**Background:**

Nerve Growth Factor (NGF) holds a great therapeutic promise for Alzheimer's disease, diabetic neuropathies, ophthalmic diseases, dermatological ulcers. However, the necessity for systemic delivery has hampered the clinical applications of NGF due to its potent pro-nociceptive action. A “painless” human NGF (hNGF R100E) mutant has been engineered. It has equal neurotrophic potency to hNGF but a lower nociceptive activity. We previously described and characterized the neurotrophic and nociceptive properties also of the hNGF P61S and P61SR100E mutants, selectively detectable against wild type hNGF. However, the reduced pain-sensitizing potency of the “painless” hNGF mutants has not been quantified.

**Objectives and Results:**

Aiming at the therapeutic application of the “painless” hNGF mutants, we report on the comparative functional characterization of the precursor and mature forms of the mutants hNGF R100E and hNGF P61SR100E as therapeutic candidates, also in comparison to wild type hNGF and to hNGF P61S. The mutants were assessed by a number of biochemical, biophysical methods and assayed by cellular assays. Moreover, a highly sensitive ELISA for the detection of the P61S-tagged mutants in biological samples has been developed. Finally, we explored the pro-nociceptive effects elicited by hNGF mutants *in vivo*, demonstrating an expanded therapeutic window with a ten-fold increase in potency.

**Conclusions:**

This structure-activity relationship study has led to validate the concept of developing painless NGF as a therapeutic, targeting the NGF receptor system and supporting the choice of hNGF P61S R100E as the best candidate to advance in clinical development. Moreover, this study contributes to the identification of the molecular determinants modulating the properties of the hNGF “painless” mutants.

## Introduction

Nerve Growth Factor (NGF) is a neurotrophin that acts on peripheral and central neurons, including sympathetic and sensory neurons and cholinergic neurons of the basal forebrain [1–4]. In addition to its neuronal targets, NGF has been shown to act on a number of non-neuronal targets, including several brain cells such as astrocytes, oligodendrocytes and microglia, cells of the immune system, blood vessel endothelial cells and many others [1, 5–8]. Due to its crucial actions during development and in adult tissues, and thanks to its pleiotropic properties, NGF holds a great and well validated therapeutic promise. In particular, its potential for clinical applications has been demonstrated for neurodegenerative disease, such as Alzheimer's disease, diabetic neuropathies, ophthalmic diseases and dermatological ulcers [9–17].

Notwithstanding the great therapeutic potential, the clinical applications of NGF have been hampered by its physiologically relevant pro-nociceptive effects [11, 18, 19]. This feature has severely limited its clinical efficacy as shown by previous clinical trials. In diabetic neuropathy clinical trial, the systemically delivered doses of NGF had to be reduced below the pharmacologically effective dose, because of the strong pain induced in patients [18]. In addition, when NGF was intra-cerebroventricularly injected into Alzheimer’s patients, the severe pain induction called for the interruption of the trial [11].

The R100W point mutation in mature hNGF is linked to the rare human genetic disease (Hereditary Sensory Autonomic Neuropathy Type V—HSAN V) [20]. In HSAN V patients, this mutation in the *NGFB* gene (exon 3, nt C661T) determines the complete loss of pain perception, without affecting most neurological functions [21].

Inspired by the HSAN V mutation in the *NGFB* gene, we developed a "painless" form of NGF, namely the mutant hNGF R100E. hNGF R100E maintains, in a variety of cellular assays, identical neurotrophic and neuroprotective properties as the hNGF wild-type, while displaying a significant reduced pain-inducing activity *in vivo* [22].

We also reported on the hNGF P61S mutant, characterized by the replacement of the Proline residue at position 61 of hNGF wild-type with a Serine residue, found in this position in mouse NGF. hNGF P61S ‘‘tagged” molecules are selectively detectable against wild type hNGF, by the monoclonal antibody 4GA which specifically recognizes hNGF P61S against the background of wild-type hNGF [23].

Finally, we have also expressed and studied the double mutant, hNGF P61SR100E, which harbours both features: the painless activity in addition to the tagging point mutation, for its potential traceability in human biological samples. In fact, the hNGF P61SR100E double mutant displays a full neurotrophic and neuroprotective activity, while it shows a reduced nociceptive activity *in vivo*, due to selective alteration of TrkA *versus* p75NTR receptor mediated binding and signalling [24, 25].

By having in mind their prospective therapeutic use in a clinical setting, the focus of this study was a thorough comparative analysis of the different mutants of hNGF, namely hNGF R100E, hNGF P61S and hNGF P61SR100E mutants, as precursor and mature forms, based on their biochemical and biophysical properties, traceability and *in vivo* pain induction activity.

## Materials and Methods

### Heterologous E. coli expression and purification of wild-type hNGF and its mutants

hproNGF wild type (WT) and the mutants were expressed as recombinant proteins in *E*. *coli*, refolded from inclusion bodies, purified, and proteolyitically processed to NGF, as previously described [26]. The proteins manufacturing method has been optimized for a scale-up production from the 1–2 to the 10 liters/batch.

### Kinetics of Proteolytic Cleavage

In order to assess the impact of the mutations on the proteolytic cleavage, studies on hproNGF WT, hproNGF P61S, hproNGF R100E and hproNGF P61SR100E were undertaken. The non-specific protease trypsin was used, that, as previously reported, cleaves the more accessible pro-peptide, and leaves NGF undigested under controlled conditions [26, 27]. 110 μg of hproNGF WT, hproNGF P61S, hproNGF R100E, hproNGF P61SR100E, at the concentration of 0.6 μg/ml in Sodium Phosphate buffer 50 mM pH 7, were proteolytically digested by trypsin (Promega Corporation, Madison, USA) at 4°C. The reaction was started adding trypsin at the ratio of 1:250 (enzyme: substrate). 15 μl of the reaction mixtures were taken at time 0, and after 0.5, 1, 1.5, 2, 3, 4, 6, 20 hours. For each sample, the reaction was blocked by addition of Laemmli sample buffer and boiling (10 minutes). All the samples were analyzed by SDS-PAGE. The experiment was repeated twice on independent samples.

### Circular Dichroism (CD) measurements

Quantitative analysis of the far-UV CD spectra provided an estimation of the secondary structural composition of the various hproNGF and hNGF mutants. CD measurements were carried out with a JASCO J-810 circular dichroism instrument at 20°C in 50 mM Sodium Phosphate, pH 7.0. Far-UV CD (185–250 nm) spectra were recorded at protein concentrations of 0.5–1.0 mg/mL in a 0.02 cm demountable quartz cuvette cell, averaged over 8 accumulations (acquisition time: 1 s). Spectra were buffer corrected. Mean ellipticity values were calculated as previously reported [26].

### Chemical denaturation

In order to characterize the effect of the mutations, the chemical stability of hNGFs and hproNGFs WT and mutants, was assessed. The changes in intrinsic fluorescence emission spectra of the hNGF WT and mutants, as a function of the denaturant concentrations, were evaluated by incubating the proteins in serial dilutions of 8 M Gdm-Cl (Guanidinium Chloride) in buffer Sodium Phosphate 50 mM pH 7. The samples were incubated at room temperature (RT) for 20 hours. The protein concentration was 20 μg/mL for hNGFs, and 40 μg/mL for hproNGFs. The proteins solutions were prepared in the native buffer (Sodium Phosphate 50 mM pH 7) or in the different concentrations of denaturing buffer (0.5–1–1.5–2–2.5–3–3.5–4–4.5–5–5.5–6 M Gdm-Cl in 50 mM Sodium Phosphate, pH 7).

Fluorescence measurements were performed, with the EnSpire Multimode Plate Reader Spectrometer Perkin Elmer (Waltham, Massachusetts, USA). The measurements, in duplicate, were done in Optiplate 96 well plate (Perkin Elmer, Waltham, Massachusetts, USA), containing 100 μl of protein solution per well. The fluorescence emission spectra were recorded from 300 to 500 nm, at a speed of 1 nm/s, with 100 flashes, using an excitation wavelength of 280 nm. The experiment was repeated three times for each protein.

All spectra were corrected against the blank spectrum of the corresponding buffer. The data were normalized, in order to compare the different hNGF mutants. The relative fraction of native hNGF molecules or the native, mature part of hproNGF, were obtained according to the following formula:

$$\alpha =\frac{\left(\text{I}-{\text{I}}_{\text{D}}\right)}{\left({\text{I}}_{\text{N}}-{\text{I}}_{\text{D}}\right)}$$

Where:

I = fluorescence signal at a certain Gdm-Cl concentration.

I_D_ or I_N_ = signal of the denatured or native component at the same Gdm-Cl concentration, respectively

I_D_ or I_N_ were calculated from the linear dependence of the fluorescence of the denatured or native protein from the concentration of the denaturing medium.

### Thermal Denaturation

The thermal denaturation of the mutants by Differential Scanning Fluorimetry (DSF—Thermofluor) was also investigated. Solutions composed of 7.5 μl of 300 x Sypro Orange (Molecular Probes, Life Technologies, Carlsbad, California, USA) and 17.5 μl of proteins (hNGFs or hproNGFs) at the concentration of 0.7 mg/ml in Sodium Phospate 50 mM pH 7, were added to the wells of a 96-well thin-wall PCR plate (Bio-Rad, Hercules, California, USA). Buffer and Water was added as blank and control sample, respectively. The plates were sealed with Optical-Quality Sealing Tape (Bio-Rad, Hercules, California, USA) and heated in an iCycler iQ Real Time PCR Detection System (Bio-Rad, Hercules, California, USA) from 20 to 90° C in increments of 0.2°C/20 s. Fluorescence changes in the wells of the plate were monitored simultaneously with a charge-coupled device (CCD) camera. The wavelengths for excitation and emission were 485 and 535 nm, respectively. The experiment was repeated three times for each protein.

In order to compare the transition curves for the set of different proteins, each experimental set of data was normalized to the transition amplitude that is the difference between the fluorescence intensity at the start and at the maximum of the unfolding transition, respectively [28].

Data were evaluated by pairwise Student’s T-tests.

### Surface Plasmon Resonance (SPR)

In order to gain further insight into the impact of the mutations on the properties of NGF, the binding profiles of the mutants against a panel of antibodies, used as structural probes, were characterized by Surface Plasmon Resonance. The binding to TrkA and p75^NTR^ receptors have been already investigated [25].

The experiments were performed with a Biacore 2000 equipment (GE healthcare, Buckinghamshire, UK). In all the cases, the experiments were performed on CM5 chips with amine coupling. The coupling reaction was performed with the specific kit provided by GE healthcare (Buckinghamshire, UK), according to manufacturer’s instructions. The antibodies (anti proNGF MAb clone EP1318Y, (Millipore, Darmstadt, Germany), anti NGF MAB 256 (R&D System, Minneapolis, Minnesota, USA), and anti NGF αD11 [29]) used as ligands were immobilized at a 2000 RU surface concentration of the CM5 chip. The analyte proteins used in all kinetic experiments were injected in PBS (Phosphate Buffer Saline) added by 5% BSA, and at a flow rate of 30 μl/min. The regeneration of the chip was performed in all the cases with a pulse (10 μl) of 10 mM Glycine pH 1.5. The data analysis was carried out using the BIAevaluation 3.2 Software by Biacore. A 1:1 Langmuir model was assumed, for the evaluation of the kinetic and equilibrium constants using the BIAevaluation software.

### TF1 Proliferation assay

In order to assess the bioactivity of NGF on human receptor TrKA, hNGF WT and mutants were tested using a TF1 erythroleukemic cells-based proliferation assay. Indeed, TF1 erythroleukemic cells express human TrKA and not p75^NTR^ [30]. The original protocol by Chevalier et al. [30], modified by Covaceuszach et al. [23], has been further optimized, as described below.

Before the assay, TF1 cells were cultured for 1 week, in RPMI-1640 containing 10% fetal bovine serum (FBS) with 2 ng/ml rhGM-CSF (R&D System, Minneapolis, Minnesota, USA). Cells for testing were washed, resuspended in RPMI-1640 + 10% FBS to a concentration of 300,000 cells/ml and replated on 96-well microplates (15,000 cells per well, in 50 ml)

60 min after replating, cells were exposed to hNGF WT and mutants (concentration range: 0.1–200 ng/ml) in RPMI-1640 containing 10% FBS. Control wells were included, either containing medium alone, or containing TF1 cells in the absence of NGF (cellular blank). Each treatment was performed in duplicate.

After a 40 h incubation period, at 37°C, 5% CO2, the medium was changed (50 μl/well RPMI-1640 containing 10% FBS.

The reagent “CellTiter 96 Aqueous One Step Solution Reagent” (Promega Corporation, Madison, USA) was thawed for approximately 90 minutes at room temperature, or 10 minutes in a water bath at 37°C. 20 μl of reagent were pipetted into each well of the 96well plate containing the cells in 50 μl of fresh culture medium.

The plate was incubated at 37°C for 1–3 hrs in a humidified, 5% CO_2_ atmosphere.

The absorbance at 490nm was recorded using an ELISA Reader (Bio-Rad, Hercules, California, USA) after 1-2-3 hrs.

The assay for each hNGF WT and mutant was repeated for 5 times, in independent experiments, and with different batches of proteins.

The assay generated a dose-response curve, which was interpolated according to the following formula:

$$\text{H}=\frac{\left({\text{H}}_{\text{max}}\times {\text{C}}_{\text{NGF}}\right)}{\left(\text{C}50+{\text{C}}_{\text{NGF}}\right)}$$

where:

H is Optical density at 490 nm; Hmax represents the maximum OD reached when the curve is at saturation; C_NGF_ is the concentration of hNGF mutants; C50 represents the concentration of hNGF determining half of the maximum effect on cell proliferation (1/2 Hmax). The data were tested by pairwise Student’s t-test, in all possible combinations, with FDR (False Discovery Rate) multiple testing correction.

### In vitro stability test by TF1 Proliferation assay

In order to further assess the stability of hNGF and mutants, the loss of bioactivity after incubation in different conditions was evaluated.

hNGF WT and the mutants were incubated at different temperature (4°C, 22°C) with the following time-course:

- Incubation at 4°C for 1 week or 2 weeks or 4 weeks
- Incubation at 22°C for 1 week or 2 weeks or 4 weeks

Aliquots of 1.5 μg of each protein at the concentration of 0.65 mg/ml were prepared. The incubations were made by using a temperature controlled water bath.

Loss of bioactivity was also evaluated after freeze and thaw cycles, and after lyophilisation. In order to carry out the cycles of freeze-thawing (5 or 12 cycles), the frozen aliquots were thawed and left at room temperature, until the solutions appeared completely unfrozen. They were refrigerated again in a -20°C fridge for 20 minutes.

The lyophilisation was made using a lyophilizator (Pirani 1001, Edwards, Crawley, England), according to manufacturer’s instructions. The lyophilized proteins were resuspended before performing the bioassay.

The *in vitro* proliferation bio-assay was carried out on TF1 erythroleukemic cells in order to evaluate the bioactivity. The hNGF WT and mutants, subjected to the previously described treatments, were always assayed in the presence of the corresponding untreated hNGF as a control.

The experiments on hNGF WT and mutants were carried out from two to six times.

The dose response curves obtained by TF1 proliferation assay were interpolated as described in the previous paragraph, and used to derive the Hmax and C50 parameters. The loss of stability mostly affected the C50 parameter. Indeed, the TF1 curves were shifted to higher values of hNGF concentration resulting in half of the maximum effect on cell proliferation (1/2 Hmax).

The ΔC50 for the different treatments were calculated, using the following formula:

$$\Delta \text{C}50=\frac{\left({\text{C}50}_{\text{treated}}-{\text{C}50}_{\text{ref}}\right)}{{\text{C}50}_{\text{ref}}}$$

C50treated is the value of C50 obtained from the curve of the protein incubated in the different conditions, C50ref is the value of C50 of the curve corresponding to the untreated hNGF. The errors were calculated based on the error propagation formulae. The data were tested by pairwise Student’s t-test, in all possible combinations, with FDR (False Discovery Rate) multiple testing correction.

### Primary culture of rat oligodendrocytes progenitor cells (OPCs) and differentiation assay

To compare the bioactivity of hNGF mutants to hNGF WT selectively through the neurotrophin receptor p75^NTR^, purified primary rat oligodendrocytes cultures (OPCs), which express p75^NTR^, but not the TrkA receptor [22], have been used. Purified cultured rat OPCs primary cultures were prepared from brain cortices of postnatal day 1 Wistar rats by mechanical dissociation as described [31]. Cells were grown on poly-D-lysine coated T75 flasks in Dulbecco’s modified Eagle’s medium (DMEM) supplemented with 20% fetal bovine serum (FBS), 4 mM L-glutamine, 1 mM Na-pyruvate, 100 U/ml penicillin, 100 U/ml streptomycin (all products are from EuroClone, Milano, Italia). After 5–6 days in culture, OPCs growing on top of a confluent monolayer of astrocytes were detached by 3–4 h horizontal shaking at 37°C. Contaminating microglial cells were eliminated by a 1 hour pre-shake and by further plating detached cells on plastic culture dishes for 1 hour. OPCs were then collected by gently washing the dishes and seeded at a density of 4 x 10^4^ cells/cm^2^ onto poly-DL-Ornithine-coated 13 mm-diameter glass coverslips placed in 24 multiwell chambers. This method yields an almost pure population (>99%) of cells of the oligodendrocyte lineage, as no contaminating microglial or neuronal cells and a very low percentage (<1%) of astrocytes were detected in these cultures when Iba1, NeuN or glial fibrillary acid protein (GFAP) immunofluorescence staining, respectively, were performed (data not shown). OPC cultures were maintained in Neurobasal medium (Life Technologies, Carlsbad, California, USA) containing 2% B27, 4 mM L-glutamine, 1 mM Na-pyruvate, 100 U/ml penicillin, 100 U/ml streptomycin, 10 ng/ml platelet derived growth factor (PDGF-BB) and 10 ng/ml basic fibroblast growth factor (bFGF; both growth factors were from PeproTech EC Ltd, London, UK) to promote cell proliferation (proliferating medium: PM). After 2–3 days in PM, cells were switched to a Neurobasal medium lacking growth factors (differentiating medium: DM) in order to allow cell differentiation.

It is known that OPCs, either in culture or *in situ*, undergo different steps of maturation identified by specific markers [32–34]. Bipolar NG2+ cells are immature OPCs which undergo differentiation to become multipolar, post-mitotic pre-oligodendrocytes and acquire O4+ immunoreactivity. After 3 days in DM, cell media were added with hNGF, hNGF R100E, hNGF P61S and hNGF P61SR100E (150 ng/ml) and incubated for further 24 h [35], before immunocytochemistry and viability test.

### Immunocytochemistry and viability test

Cells were characterized for developmental-dependent antigen expression by using the NG2 and O4 monoclonal antibodies. At the end of treatment, purified primary OPCs were fixed with 4% paraformaldehyde in 0.1 M PBS for 20 minutes at RT. The following primary antibodies were diluted in bovine serum dilution buffer (BSDB: NaCl 450 mM, Sodium Phosphate 20 mM, pH 7.4, 15% bovine serum, 0.3% Triton X-100) and incubated for 2.5 h at RT: mouse anti-NG2 (Millipore, Darmstadt, Germany; 1:500) or mouse anti-O4 (Millipore, Darmstadt, Germany). Cells were then washed three times with PBS and incubated for 1 h at RT with donkey anti-mouse secondary antibody (diluted 1:500 in BSDB) conjugated to AlexaFluor 555 (Molecular Probes, Life Technologies, Carlsbad, California, USA). Coverslips were mounted with Vectashield mounting medium (Vector Laboratories, Cambridgeshire, UK) containing 4′,6-diamidino-2-phenylindole (DAPI) to visualize cell nuclei, and analyzed by using an Olympus BX40 microscope coupled Image Analysis Software (Olympus, Tokyo, Japan). Preliminary negative control experiments, performed by omitting primary antibody and incubating fixed cells with the secondary antibody alone, were made for each condition in order to exclude non-specific binding (data not shown). The number of cells labeled by a specific antibody (mouse anti-NG2 and mouse anti-O4) in each coverslip was quantified by counting the number of fluorescent cells in 10 random microscopic fields (20x) and expressed as a percentage over the total cell number (DAPI + nuclei) in the same field. In each experimental session three coverslips for any different condition were evaluated. At least 3 experimental sessions were performed.

Cell viability was assessed through the 3-(4,5-dimethyl thiazol-2-y1)-2,5-diphenyl tetrazolium bromide (MTT; Sigma-Aldrich, Saint Louis, Missouri, USA). Briefly, cells were plated in a 96 multiwell (10^3^ cells/well) and treated with wild type or mutants at 150 ng/ml, 24 hours). MTT was added during the last hour of incubation. The medium was then removed and 100 μl of DMSO added to each well to dissolve the dark blue crystals. Microplates were then read on microplate reader, using a test wavelength of 550 nm. At least six wells for any given experimental condition were tested. Experiments were run in triplicate.

### ELISA system to detect P61S tagged hNGF mutants

In order to selectively detect, in biological fluids, the mutants containing the P61S point mutation from the endogenous hNGF, an ELISA assay has been developed, exploiting the anti-NGF antibody 4GA [23]. A classical sandwich format has been designed: the anti-NGF 4GA antibody [23] was captured on the plastic wells, recombinant human hNGF P61S was used for a standard calibration. The rabbit anti-NGF polyclonal antibody H-20 (sc-548, Santa Cruz, Santa Cruz, California, USA) was used as detecting antibody. The secondary antibody was an HRP conjugated anti-rabbit IgG (Minimum Cross Reactions, Jackson laboratories, West Grove, Pennsylvania, USA). The assay was carried out by using 4% of milk (Sigma-Aldrich, Saint Louis, Missouri, USA) in blocking and sample buffers (4% milk in PBS).

The concentrations and the incubation times of the reagents are as follows:

- Anti-NGF 4GA antibody: 2 μg/ml, coated overnight at 4°C in Carbonate buffer;
- Blocking buffer: 4 hours at room temperature;
- Calibration curve and samples: overnight at 4°C (duplicates);
- Primary antibody: concentration 1:200, 6 hours at room temperature;
- Secondary antibody: concentration 1:7000, 1 hour at room temperature.

The reaction was revealed using TMB (Sigma-Aldrich, Saint Louis, Missouri, USA), which gave rise to a colorimetric reaction in 20 minutes. The reaction was stopped by the addition of sulphuric acid 1 M and the Optical Absorbance was read at 450 nm by an ELISA reader (Bio-Rad, Hercules, California, USA).

In order to confirm the specificity of the assay and the absence of cross-reactivity between the hNGF P61S or hNGF P61SR100E mutants and wild-type NGF from various species, the calibration curve was also carried out using mouse NGF, rat NGF and human NGF as calibrators.

In order to assess the applicability of the ELISA to measure the hNGF P61S mutant into biological samples, some validation tests were carried out on homogenized brain tissues, from wild-type mice and rats. In the case of the mouse samples, the ELISA assay was carried out on a pool of animals, while in the case of rats, single animals were individually analyzed, and signals were averaged. Animal experiments were carried out according to Italian legislation (DL 116/92) and European Communities Council Directive (86/609/EEC). The tissues from mice are dissected from the wild type strain described in Tiveron et al.[36]. The experiments with rats were conducted under the permit (number CBS-1611, Scuola Normale Superiore, Pisa) approved by the Italian National Committee for animal research.

In order to evaluate the background effect of biological matrixes, two different concentrations of the recombinant hNGF P61S mutant were spiked in the mice and rat brain tissues. For each assay, the samples were analyzed in duplicate and the measured values were compared with the theoretical values expected from the spiking, to obtain the recovery percentage.

The test was performed by different operators (10 assays), in order to assess the reproducibility of the assay.

The calibration curve was also carried out by using hNGF P61SR100E as calibrator, and the assay was performed as described above, in order to validate the assay also with the double mutant.

### In vivo study of the nociceptive effects

Animal experiments were carried out according to Italian legislation (DL 116/92) and European Communities Council Directive (86/609/EEC). Studies were conducted under the permit (number 143/2008-B, University of Florence) approved by the Italian National Committee for animal research. CD1 mice (male, 25–30 g, Harlan Laboratories, Indianapolis, Indiana, USA) were used for nociceptive tests. Mice were housed in a temperature- and humidity-controlled vivarium (12 h dark/light cycle, free access to food and water). Behavioral experiments were performed in a quiet, temperature-controlled room (20 to 22°C) between 9 a.m. and 5 p.m. by an operator blinded to the status of drug treatment. Different groups of mice were used for mechanical allodynia and hot hyperalgesia behavioral measurement. At the end of the experiments, animals were sacrificed with a high dose of intraperitoneal (i.p.) sodium pentobarbital (200 mg/kg). For each experimental group 4–6 mice have been assigned. Experiments were run in triplicate.

Mice were injected i.pl. with 20 μl of different doses of hNGF WT (0.1, 1, 4 and 10 μg/paw) or hNGF R100E, hNGF P61S and hNGF P61SR100E mutants (all 1, 4 and 10 μg/paw) diluted in isotonic saline (0.9% NaCl) and the pro-nociceptive effects of hNGF WT were compared to that of hNGF R100E, hNGF P61S and hNGF P61SR 100E mutants. Control mice were injected with 20 μl of isotonic saline. Behavioral measurements were performed before (baseline, BL) and 1, 3 and 5 hours after i.pl. injection for mechanical allodynia and 1, 3 and 5 hours after i.pl. injection for hot hyperalgesia.

#### Mechanical allodynia

Mechanical allodynia was quantified as paw withdrawal threshold in response to a mechanical stimulus of increasing strength, using manual von Frey filaments (Ugo Basile, Varese, Italy) and the up-and-down paradigm as previously described [37]. Briefly, mice (6–8 for each experimental group) were placed in transparent Plexiglas chambers (30x30cm) with a wire net floor, 30 minutes before the experiment. Mechanical nociceptive threshold was determined before (basal level threshold, BL) and after (1, 3, 4, and 5 hours) different treatments. The 50% mechanical paw withdrawal threshold (in g) response was then calculated from these scores, as described [38].

#### Thermal hyperalgesia

Hot hyperalgesia was assessed in mice before (BL) and at selected time points (1, 3 and 5 hours) after i.pl. injection of wild type or mutant hNGF by placing animals on a hot plate (Ugo Basile, Varese, Italy) with temperature adjusted to 50 ± 0.1°C [39]. The latency to the first hind paw licking or withdrawal was taken as an index of nociceptive threshold. The cut-off time was set at 30 seconds, to avoid paw damage. The paw withdrawal latency to the first response was reported as mean of two different trials.

## Results

### E. coli expression and purification of wild-type hNGF and its mutants

All the analysis reported here were performed on the short form of recombinant human proNGF, namely hproNGF25 (according to the nomenclature in Paoletti et al. [27], see S1 Fig). For the sake of brevity, throughout the manuscript the protein is simply named as hproNGF, followed by the specific mutation.

hNGF WT and its mutants were produced in *E*.*coli* as recombinant proteins, and purified after proteolytic cleavage from their corresponding proNGF precursors, as described [26, 27]. The expression and growth conditions were scaled up. The purity for hNGF and for all the mutants was estimated to be at least 95% by SDS-PAGE overloading. The scaling-up procedure delivered higher yields for almost all the mutants (Table 1).

**Table 1:** Yield (mg of protein/Liter of culture) of NGF and proNGF after scaling-up process.

Protein	Scale of culture(L)	Yield ProNGF (mg/L)	Yield NGF (mg/L)
hNGF	10	35	16
hNGF P61S	10	24	12
hNGF R100E	10	9.3	1.6
hNGF P61S R100E	10	8.3	3.5

### Kinetics of Proteolytic Cleavage

Proteolytic studies on hproNGFs (hproNGF WT, hproNGF P61S, hproNGF R100E and hproNGF P61SR100E) were undertaken, in order to detect possible differences in the stability of the mutants and to assess whether the R100E mutation affects the proteolytic cleavage. Different proteases are known to cleave proNGF [40, 41]. In the present study, the unspecific protease trypsin was used, that, as previously reported, cleaves the more accessible pro-peptide, and leaves NGF undigested under controlled conditions [26, 27].

**Fig 1:**
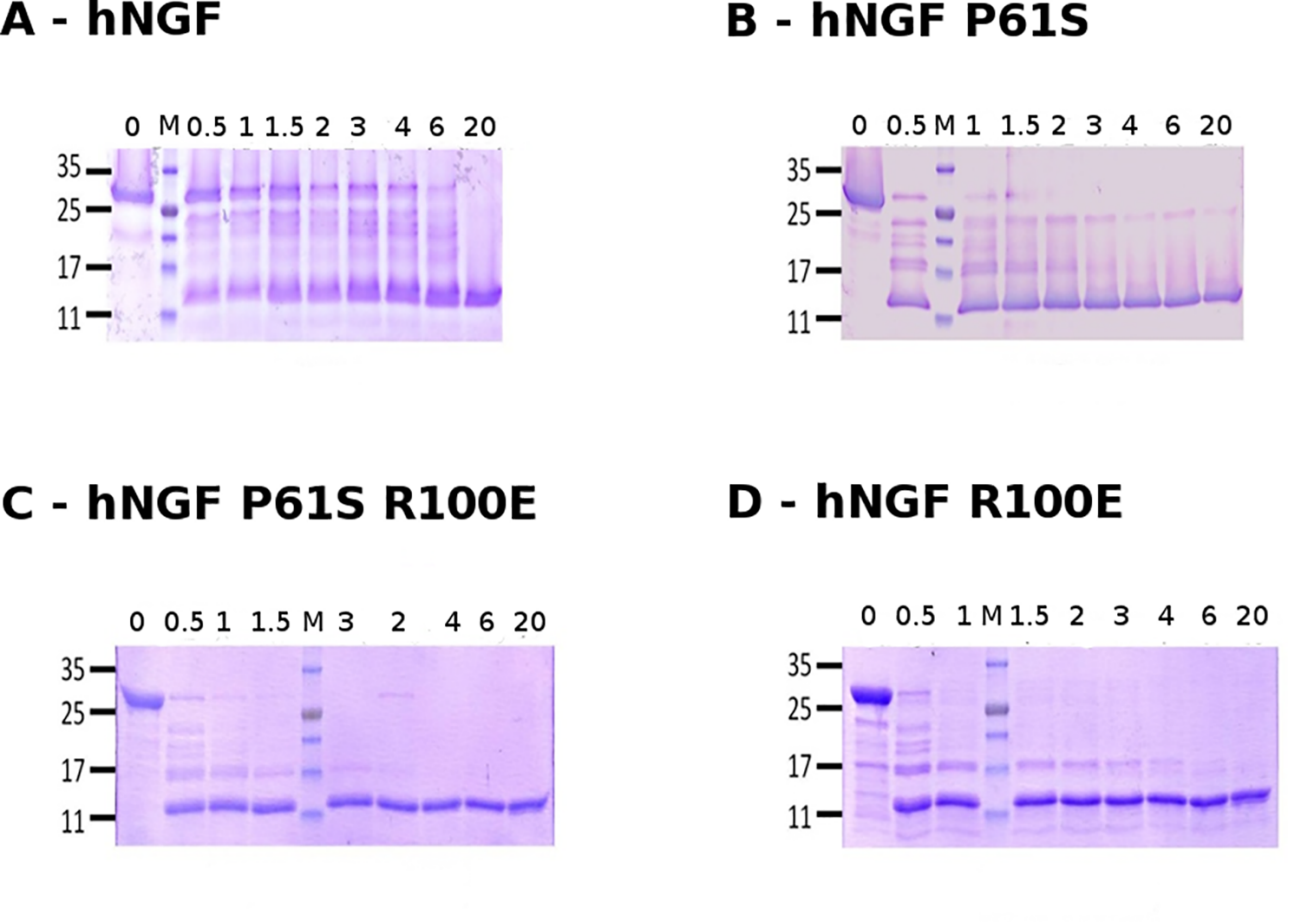
Kinetics of proteolytic cleavage of proNGF WT and mutants. Representative SDS-PAGE of hproNGF WT (panel A), hproNGF P61S (panel B), hproNGF P61SR100E (panel C), hproNGF R100E (panel D) digested by trypsin. The lanes correspond to aliquots of the reaction mixtures, taken at time 0 (immediately after trypsin addition) and after 0.5, 1, 1.5, 2, 3, 4, 6, 20 hrs. The loading position of the molecular weight marker is indicated by M.

hproNGF WT was highly resistant to proteolytic cleavage, its digestion to NGF being completed after 20 hours. During the incubation, a decrease in intensity of the hproNGF WT band and a concomitant increase in the intensity of the hNGF one, were evident. A pattern of cleavage intermediates, with molecular weights in the 17 kDa to 25 kDa range, was also reported (Fig 1).

The hproNGF P61S digestion profile differed from that of hproNGF WT, because the bands corresponding to the precursor and to the intermediates forms, gradually decreased upon incubation time. Indeed, the hproNGF P61S band completely disappeared after 3 hours, while for hproNGF WT, the bands corresponding to the precursor and to the intermediates forms persisted after 6 hours of incubation. A 23 kDa band, that likely corresponds to a stable intermediate, was observed in the digestion profile of hproNGF P61S after 6 hours (Fig 1).

The hproNGF R100E and hproNGF P61SR100E mutants digestion profiles, showed a stable and well represented intermediate of 17 kDa which completely disappeared within 4 hours in the hproNGF R100E, and within 2 hours in the hproNGF P61SR100E digestion profiles, respectively (Fig 1). The digestion of hproNGF P61SR100E was completed after 4 hours.

The hproNGF R100E band disappeared already after 0.5 hours, with the concomitant appearance of the hNGF R100E. It is important to notice that hNGF R100E, but none of the other mature NGF proteins, was further cleaved by trypsin (Fig 1).

Overall, the different hproNGFs mutants showed different digestion kinetics and a distinct time-dependent accumulation of stable intermediates. In particular, hproNGF WT and proNGF P61S appeared to be similarly quite resistant to proteolytic cleavage, while hproNGF R100E and hproNGF P61SR100E were cleaved more rapidly, with hNGF R100E being the only mutant which digestion also proceeded on the mature form.

### Circular Dichroism (CD) studies

The overall folding of the various hproNGF and hNGF mutants was evaluated by estimating the secondary structural features from the far-UV circular dichroism spectra.

On the whole, hNGF and hproNGF mutants showed spectra in good agreement with those previously published [26, 27]. It is, however, interesting to notice some distinct features of these spectra.

**Fig 2:**
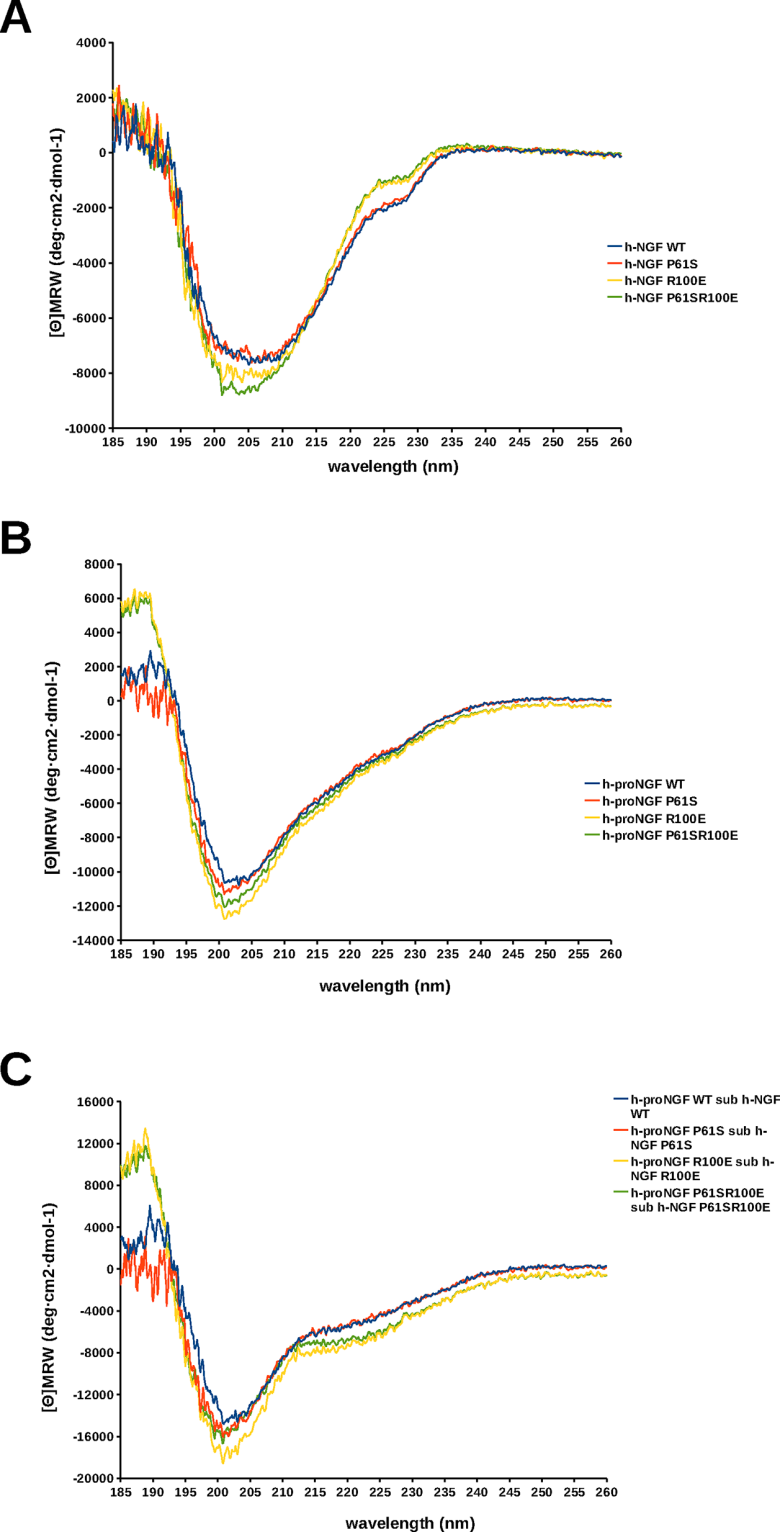
Comparison of far UV CD spectra. Far-UV CD spectra of WT (blue), P61S (red), P61SR100E (green), R100E (yellow) hNGF (panel A), hproNGF (panel B) and pro-peptide (obtained by subtracting the NGF curve from the proNGF one) (panel C).

It was apparent that the hNGF WT and hNGF P61S spectra completely overlapped, while both differed from the hNGF R100E and hNGF P61SR100E spectra. The main differences (Fig 2A) were in the position and intensity of the minimum around 202–207 nm, and in the near UV region around 225-230nm. These changes likely reflect small rearrangements in the secondary structure elements of the protein, as a result of the R100E mutation. Indeed, the charge inversion is expected to disrupt ionic and hydrogen bonding interactions with the spatially neighbouring residues.

In the case of the hproNGF mutants, the differences were less marked, although a variation in the intensity of the minimum at 200–205 nm was still visible (Fig 2B).

Interestingly, the difference spectra between the corresponding hproNGFs and hNGFs mutants, that should represent the secondary structure contribution of the pro-peptides, were not completely overlapping (Fig 2C). The difference spectra of hproNGF WT and hproNGF P61S pro-peptides almost overlapped, while both differed from the difference spectra of the hproNGF R100E and hproNGF P61SR100E mutants, that instead were very similar one to the other. This feature allowed to hypothesize that although the R100E mutation resides in the mature domain of the protein, it influences the arrangement of the whole proNGF domain. Indeed, the R100E mutation is located quite close in space to residue W21 in mature NGF that in turn has been suggested to interact with the pro-peptide [25, 27, 42].

In conclusion, it appears that the mutation P61S shows a very limited influence on the secondary structure of mature NGF, while the R100E mutation has a greater impact by likely affecting the secondary structure of the pro-peptide moiety.

### Chemical and thermal stability analysis of NGF mutants

In order to further characterize the effect of the mutations, the chemical and thermal stability of the hNGFs and hproNGFs mutants, were assessed.

**Fig 3:**
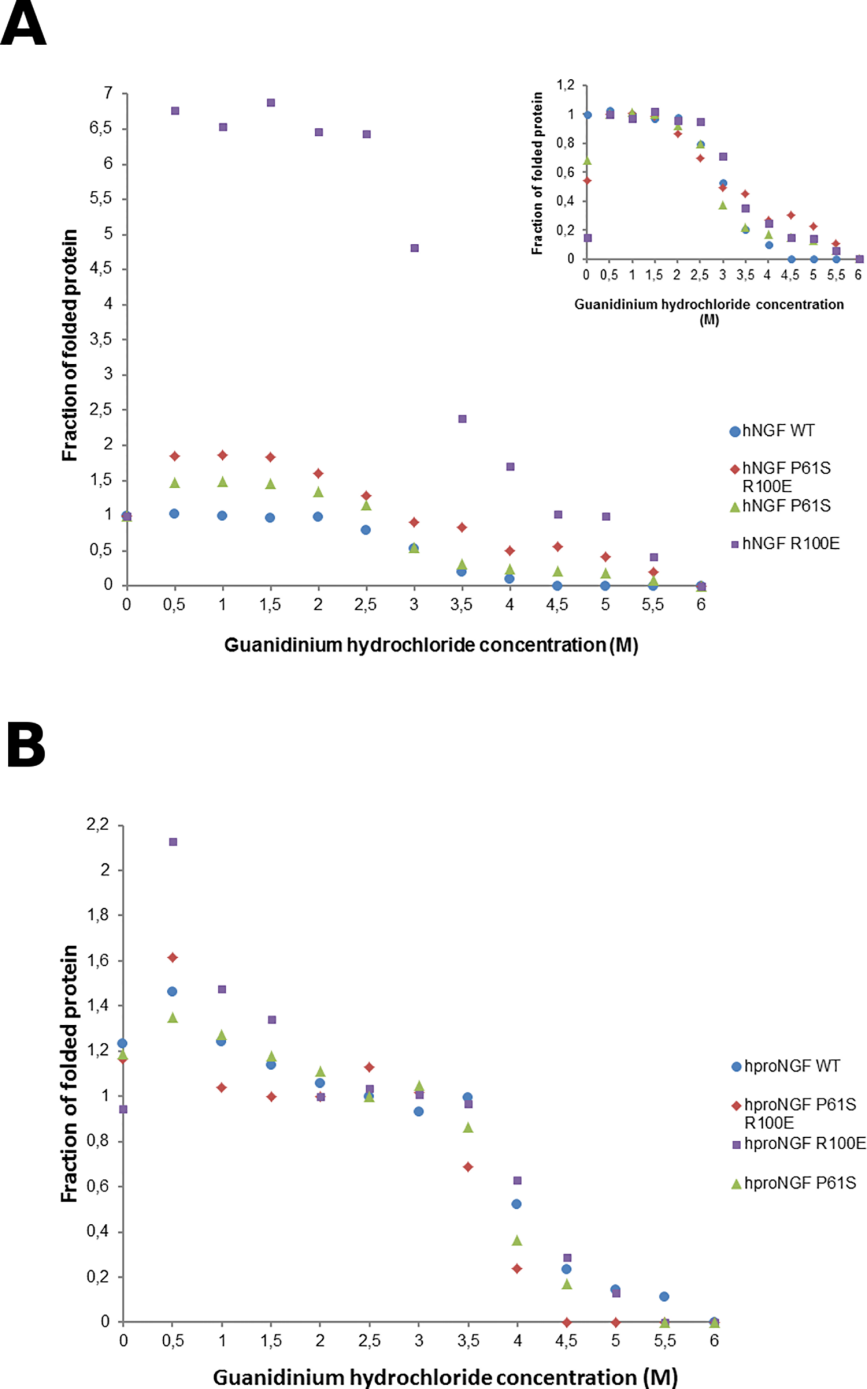
Chemical denaturation. Guanidinium Chloride (Gdm-Cl) denaturation profile of WT (blue), P61S (green), P61SR100E (red), R100E (purple) hNGF (panel A), hproNGF (Panel B). The fraction of folded protein is plotted as a function of Gdm-Cl concentration. The inset in Panel A shows an alternative normalization, obtained to consider the anomalous behaviour of the mutants that show an increase in the emission fluorescence intensity at low Gdm-Cl concentrations (0–1.5M). The data were normalized assuming that the mutants, unlike hNGF WT, are in a fully folded state (Fraction folded = 1) at 0.5M Gdm-Cl (instead of that at 0M Gdm-Cl) and allow to better compare all the proteins at a glance.

At first, the chemical denaturation was investigated. hNGFs and hproNGFs mutants, were incubated with increasing concentrations of Gdm-Cl as a denaturant, and the fluorescence emission spectra measured. The fluorescence intensity maximum changed as a function of increasing Gdm-Cl concentration (see Fig 3A and 3B for hNGFs and hproNGFs respectively–the fraction of native protein is reported as a function of the Gdm-Cl concentration).

**Fig 4:**
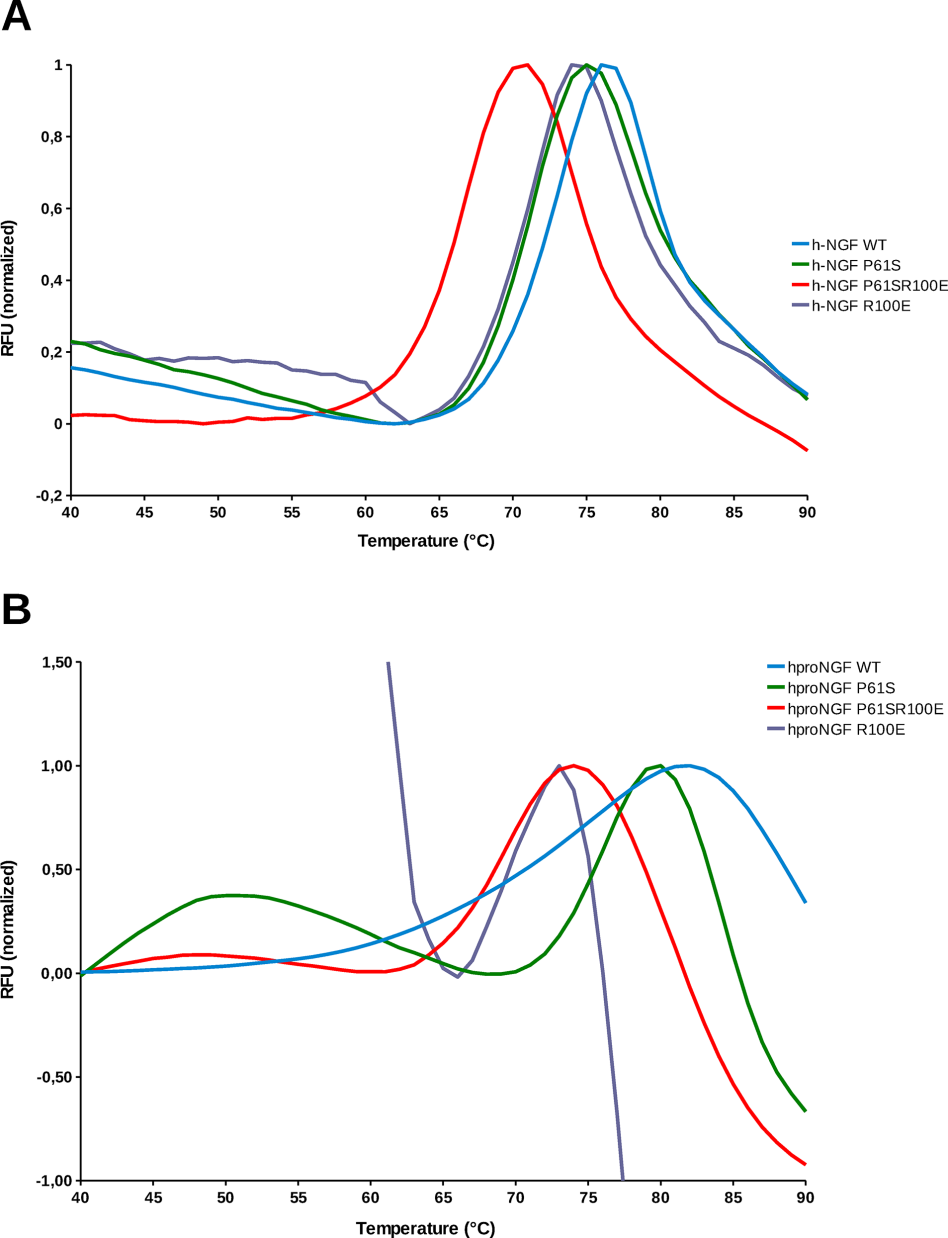
Thermal denaturation. Thermal denaturation profile of WT (blue), P61S (green), P61SR100E (red), R100E (purple) hNGF (panel A), hproNGF (panel B) measured as a thermal shift assay by Differential Scanning Fluorimetry (DSF). Normalized RFU (Relative Fluorescence Unit) is plotted in function of temperature.

hNGF WT showed the classical sigmoidal shape curve associated with a two-state unfolding. Unexpectedly, the different mutants behaved very differently. All the mutants showed initially, at low Gdm-Cl concentrations (0–1.5M), an increase in the emission fluorescence intensity, reaching higher values than those measured in the absence of Gdm-Cl (Fig 4A). Subsequently the signal decreased following a sigmoidal shape until the fully denatured state is reached (at 6M Gdm-Cl–fraction folded protein = 0). This unusual behaviour at low Gdm-Cl concentration could be explained in terms of the likely electrostatics interactions occurring between the proteins and Gdm-Cl [43–45]. Thus, at low denaturant concentration, the ionic nature of Gdm-Cl increases the stability of the protein, by masking its intra- and intermolecular electrostatic interactions, as observed for the hNGF P61S, hNGF R100E and hNGF P61SR100E mutants. This behaviour was reported for model proteins designed with intra- and intermolecular predominating electrostatic repulsive rather than attractive interactions [43]. At increasing concentrations, on the contrary, Gdm-Cl exerts its expected denaturant activity, and the intra- and intermolecular electrostatic interactions are no longer significant.

The hNGF mutants exhibited a sigmoidal shape at higher Gdm-Cl concentrations and eventually unfolded (see Fig 3A). For this reason, we also analyzed the data according to a different data normalization, where we assumed that the mutants, unlike hNGF WT, were in a fully folded state (Fraction folded = 1) at 0.5M Gdm-Cl instead of that at 0M Gdm-Cl, (see inset in Fig 3A). This representation allowed to better compare the behaviour of all the proteins at a glance. The stabilizing effect at low Gdm-Cl concentration was not significant for hNGF P61S and hNGF P61SR100E, whereas it was highly significant for hNGF R100E (Fig 3A).

Therefore, the mutations introduced in hNGF proteins have an effect on the overall electrostatic potentials of the protein, the R100E mutation showing the maximum destabilizing effect, which is partly counteracted by the concomitant presence of the P61S mutation (see Fig 3A), as apparent in the hNGF P61SR100E mutant. These effects, however, do not necessarily imply an overall protein destabilization. In fact, all the mutants showed to have a comparable point of inflection of their respective denaturation curves (Table 2).

**Table 2:** Inflection points extrapolated by the chemical denaturation profiles of human NGF and proNGF Wild Type and mutants.

	**hNGF WT**	**hNGF P61S**	**hNGF R100E**	**hNGF P61S R100E**
**inflection point (M)**	3.0	2.8	3.2	3.0
	**hproNGF WT**	**hproNGF P61S**	**hproNGF R100E**	**hproNGF P61S R100E**
**inflection point 1 (M)**	1.5	1.5	1.0	1.0
**inflection point 2 (M)**	4	3.8	4.2	3.2

The hproNGFs mutants showed a more homogeneous behaviour, with no significant changes in the curve shapes. The occurrence of two inflection points, as already reported [26, 27] reflects the denaturation of the pro-peptide as the first event, followed by unfolding of the mature moiety. For the normalization of the hproNGF curves, it was assumed that the NGF moiety is the reference, and therefore a value equal to 1 was assigned to the Gdm-Cl concentration corresponding to the plateau after the first denaturation event, corresponding to a fully folded mature NGF. The mutations did not have a significant effect on the shift in the position of the first inflection point in the hproNGF series, while they had a significant effect in the position of the second one, as shown in Table 2. It is noteworthy that for all mutants, the denaturation point corresponding to the unfolding of the mature NGF, in the context of proNGF, had a significant shift versus higher Gdm-Cl concentrations when compared to the mature hNGFs, thus confirming a stabilizing effect of the pro-peptide on the mature part of the protein, as already reported [46, 47].

The thermal denaturation of the mutants was also investigated, by Differential Scanning Fluorimetry (DSF—Thermofluor). As shown in Fig 4A, the mutations had a small although significant destabilizing effect (P<0,01) effect on the melting temperature of the hNGF proteins, when compared to the hNGF WT, with the double mutant showing a higher effect (Table 3).

**Table 3:** Melting Temperature (Tm) extrapolated by the thermal denaturation profiles of human NGF and proNGF Wild Type and mutants.

	**hNGF WT**	**hNGF P61S**	**hNGF R100E**	**hNGF P61S R100E**
**Tm (°C)**	72.1 ± 0.2	70.6 ± 0.1	70.4 ± 0.1	65.5 ± 0.6
	**hproNGF WT**	**hproNGF P61S**	**hproNGF R100E**	**hproNGF P61S R100E**
**Tm (°C)**	70.8 ± 0.1	74.9 ± 0.1	69.5 ± 0.4	68.4 ± 0.2

The values are averages of the different experiments. The errors represent standard deviations.

The R100E mutation had a destabilizing effect also on the hproNGFs mutants when compared to the hproNGF WT (P<0,01), even though smaller than in the case of the mature proteins, (Fig 4B), while the P61S mutation seemed to even have an increased stabilizing effect. Notably, not all the samples showed a double transition that likely corresponds to a two-step denaturation mechanism, *i*.*e*. the unfolding of the pro-peptide as the first event, followed by the unfolding of the mature part. The results might be apparently counterintuitive. However, it should be taken into consideration that the DSF assay makes use of the Sypro Orange dye that is known to bind to the hydrophobic surfaces of the proteins. Therefore, the highly flexible pro-peptide might be prone to interact with the dye giving rise, during the initial unfolding event, to unusual denaturation profile curves (Fig 4B).

### TrkA-dependent and p75-dependent bioassays: TF1 Proliferation assay and Oligodendrocytes differentiation assay

The receptor specific biological activity of the hNGF mutants was comparatively evaluated by two cellular assays: the proliferation assay using TF1 human erythroleukemic cells, and the differentiation assay of primary rat oligodendrocytes, in order to confirm the consequences on the biological activity of the NGF mutations on TrkA-mediated biological activity and to add knowledge on the effects of the double mutation on p75^NTR^ activity.

In order to compare the bioactivity of hNGFs on TrkA receptor, hNGF WT and hNGF mutants were tested using the TF1 erythroleukemic cells-based proliferation assay. This human cell line expresses human TrkA receptor in the absence of detectable p75^NTR^ [30]. NGF induces autophosphorylation of TrkA and substitutes for granulocyte-monocyte colony-stimulating factor (GM-CSF) to trigger the proliferation of TF1 cells. Thus, the assay provides a quantitative measure of NGF activity and potency *via* human TrKA receptors.

**Fig 5:**
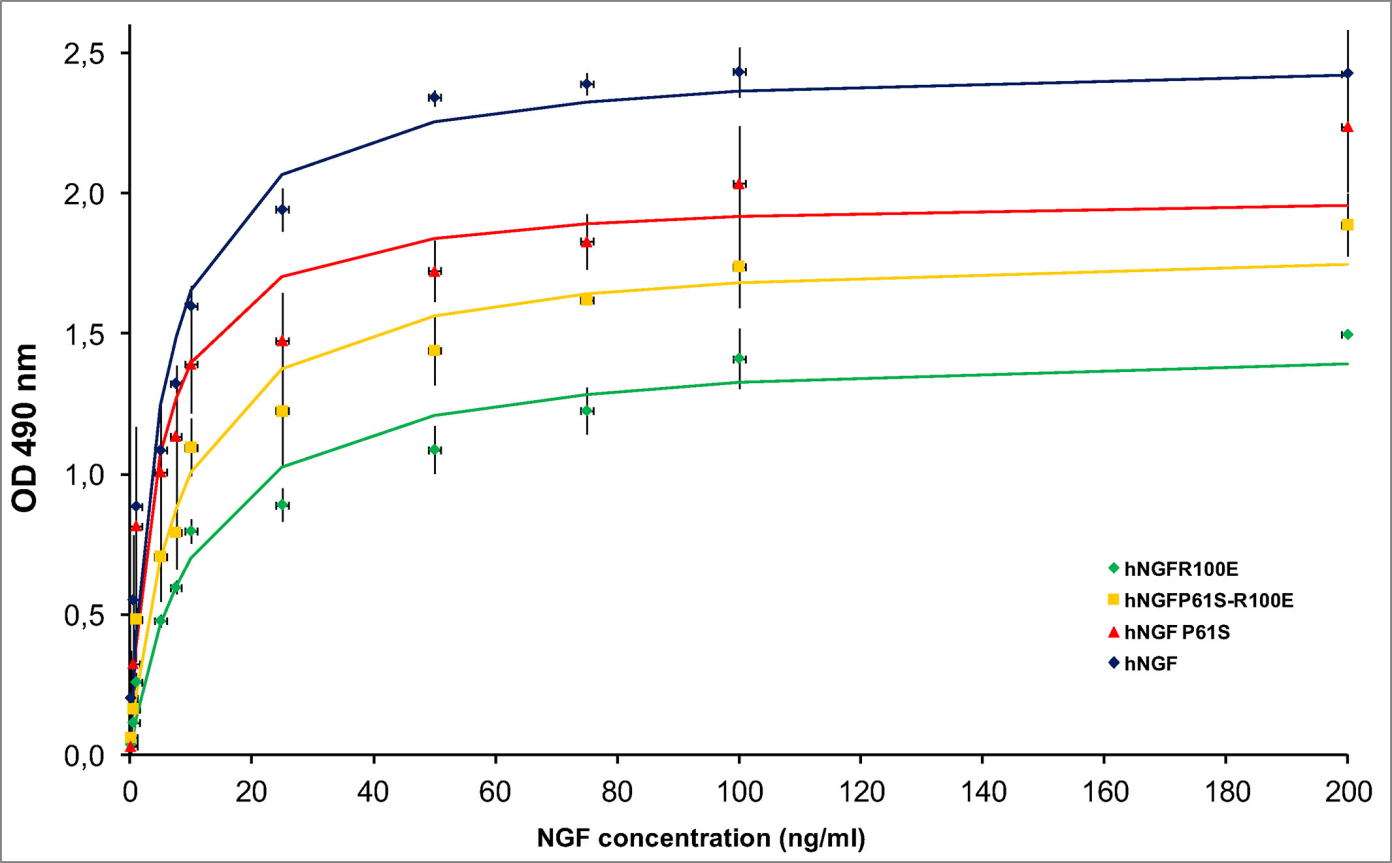
TF1 cells proliferation assay. Interpolated TF1 proliferation curves of hNGF WT (blue), hNGF P61S (red), hNGF P61SR100E (yellow), hNGF R100E (green). The indicators represent the experimental points (averages), the error bars the standard deviations, the continuous lines are the result of the interpolations.

Each hNGF protein, tested by this quantitative TF1 bioassay, exhibited a characteristic dose response curve (Fig 5). Moreover, as evident from the small error bars in Fig 5, for each mutant there was a high consistency between different protein batches and different experiments. We conclude that the TF1 bioassay can be considered a biological and quantitative “fingerprint” for each mutant.

Table 4 reports the maximal TF1 proliferation induced by the various hNGFs mutants (Hmax) and the NGF concentration determining a half maximum effect on cell proliferation (C50), calculated from the curves interpolating the experimental points. The data were tested by pairwise Student’s t-test, in all possible combinations, with FDR multiple testing correction. Hmax values exhibited a p value <0.05 for all the pairs analyzed, while the only significant difference (p<0.05) in the C50 pairs analyzed was that between hNGF P61S and hNGF R100E.

**Table 4:** Parameters extrapolated by the interpolation of the TF1 proliferation curves of human NGF wild type and mutants.

Protein	Hmax (nm)^a^	C_50_ (ng/mL)^b^
hNGF WT	2.5 ± 0.1	5.0 ± 1.3
hNGF P61S	2.0 ± 0.1	4.3 ± 1.3
hNGF R100E	1.5 ± 0.1	10.9 ± 2.1
hNGF P61S R100E	1.8 ± 0.1	8.1 ± 1.7

^a^ Hmax is the maximum OD reached when the curve is saturated.

^b^C50 represents the concentration of hNGF determining half of the maximum effect on cell proliferation.

The values represent the average of the calculated Hmax and C50 parameters. The errors are standard deviations.

hNGF WT treatment in TF1 cells resulted in a strong proliferative response. The hNGF P61S treatment yielded a response hNGF WT-alike by exhibiting a similar C50 value, but a lower Hmax value. The painless mutant hNGFR100E showed a drop in Hmax. hNGF P61SR100E exhibited an intermediate action in between those of the hNGF P61S and hNGF R100E mutants.

To compare the bioactivity of hNGF mutants to hNGF WT selectively through the neurotrophin receptor p75^NTR^, purified primary rat oligodendrocytes cultures (OPCs), which express p75^NTR^, but not the TrkA receptor [22], were used. hNGF, by activating p75^NTR^, inhibits OPCs differentiation [22, 40], therefore we investigated whether hNGF mutants were able to signal through p75^NTR^ by measuring OPCs differentiation. OPCs spontaneously differentiate from immature, proliferating, NG2+ cells, versus O4+ post-mitotic pre-oligodendrocytes. Thus, an increase in the percentage of NG2+ cells, or a decrease in the percentage of O4+ cells in comparison to control conditions, indicates inhibition of OPCs differentiation.

**Fig 6:**
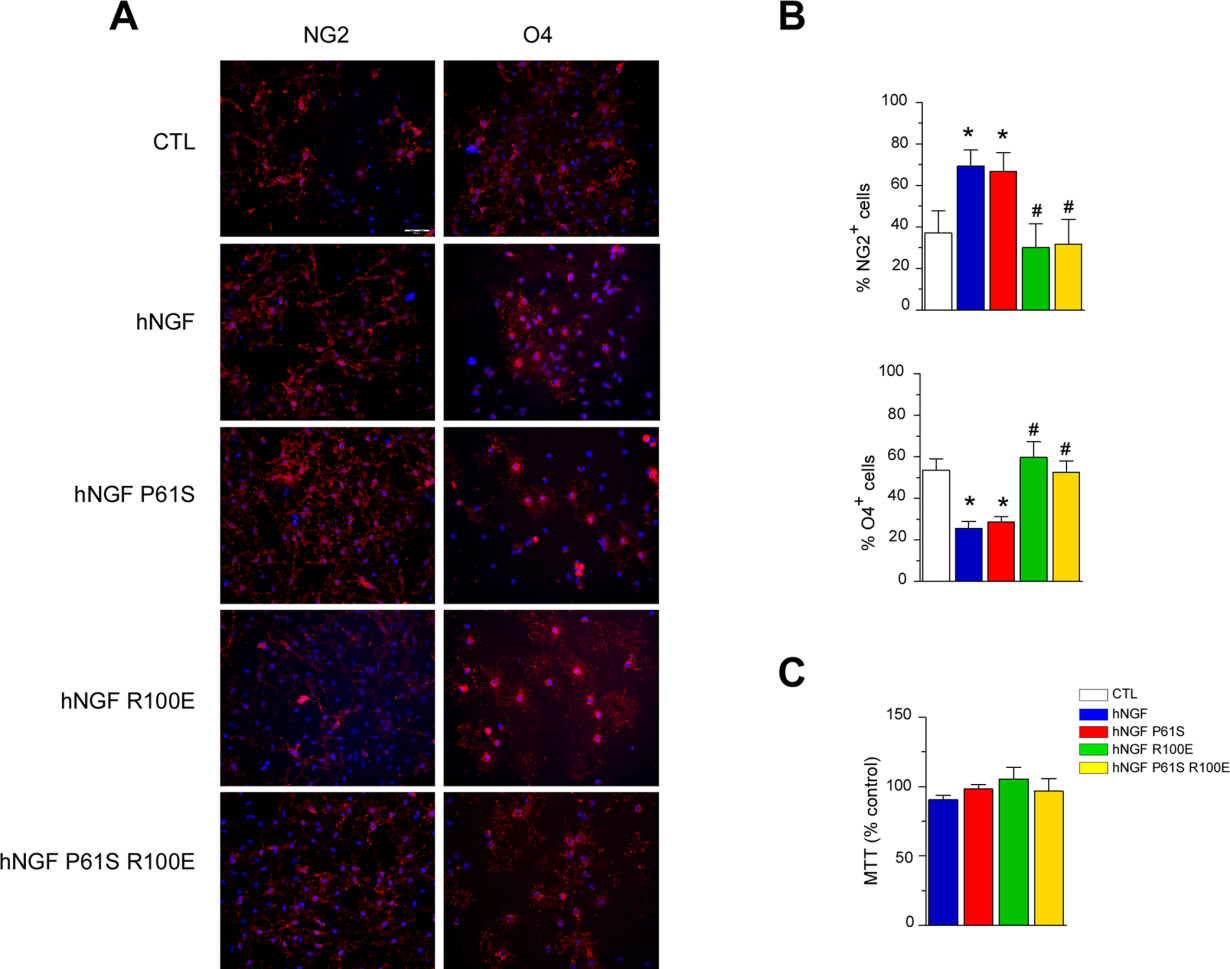
hNGF WT and mutants bioactivity on *in vitro* differentiation of purified primary rat OPCs. Panel A: Immunolabelling of primary purified OPC cultures grown for 24 hours in control conditions or in the presence of hNGF WT or its mutants (R100E, P61S, P61S R100E; all compounds are 150 ng/ml). Cell nuclei are marked with DAPI (blue). Scale bar: 50 μm. hNGF increases the percentage of undifferentiated NG2+ cells (left panels: in red) and decreases the percentage of O4+ pre-oligodendrocytes (right panels: in red), indicating that NGF inhibits OPC differentiation. The same effect is observed when cells were growth in the presence of the hNGF mutant P61S but not of R100E nor P61S R100E. Panel B. Quantification of the percentage NG2+ (upper panel) and O4+ (lower panel) OPCs in all different experimental conditions. Three coverslips per experiment were performed in each experimental group. Ten random microscopic fields (20x) per coverslip were evaluated. Experiments were run in triplicate. *P<0.05 vs CTL, #P<0.05 vs hNGF, One-way ANOVA followed by Newman-Keuls post-test. Panel C. None of the compounds tested (all 150 ng/ml; 24 hours exposure) induced toxicity in rat OPC cultures.

First, by using immunocytochemistry in rat OPC primary cultures we confirmed that exposure to hNGF WT (150 ng/ml, 24 hours) increased the percentage of undifferentiated NG2+ cells and decreased the percentage of O4+ pre-oligodendrocytes, indicating that hNGF inhibits OPCs differentiation, concurring with previous reports [22] (Fig 6). Then, we evaluated the effect of the various mutants and we observed that exposure to hNGF P61S (150 ng/ml, 24 hours) produced similar effects to hNGF WT on OPCs differentiation, enhancing the percentage of the undifferentiated NG2+ cells in comparison to the untreated control group (Fig 6). Furthermore, this inhibitory effect was absent when OPCs were grown in the presence of hNGF R100E or hNGF P61SR100E mutants (both 150 ng/ml, 24 hours) (Fig 6). In fact, as expected by their lower binding affinity for the p75^NTR^ with respect the wild-type form [25], the mutants hNGF R100E and hNGF P61SR100E were similarly inactive in inhibiting OPC differentiation since the percentage of either NG2+ or O4+ cells observed in the presence of these mutants was unchanged as compared to controls (Fig 6). MTT viability assay revealed that none of the proteins tested, either wild-type or mutants, did induce any toxicity in rat OPC primary cultures (Fig 6). Thus, the present data demonstrate that both hNGF R100E and hNGF P61SR100E mutants show a similar reduced ability to inhibit OPCs differentiation as compared to the hNGF WT.

### In vitro stability of hNGF WT and the mutants by TF1 Proliferation assay

In order to further assess the stability of WT and mutants hNGF, the loss of bioactivity following incubation of the proteins in different conditions was evaluated. This experiment was carried out also to provide indications for the experimental handling of hNGF and mutants in research activities and in a future clinical use as drugs.

hNGF WT and mutants were incubated at different temperature (4°C, 22°C) with the time-course described in the Materials and Methods section, and their bioactivity was tested using the TF1 erythroleukemic cells-based proliferation assay. Using the same biological readout, the bioactivity was also evaluated after 5 and 12 freeze and thaw cycles, and after lyophilisation.

The dose response curves obtained by TF1 proliferation assay were interpolated, and Hmax and C50 values were obtained. C50 was the parameter more affected by the loss of stability. Indeed, the TF1 curves appeared shifted to higher value of hNGF concentration determining half of the maximum effect on cell proliferation (1/2 Hmax).

**Fig 7:**
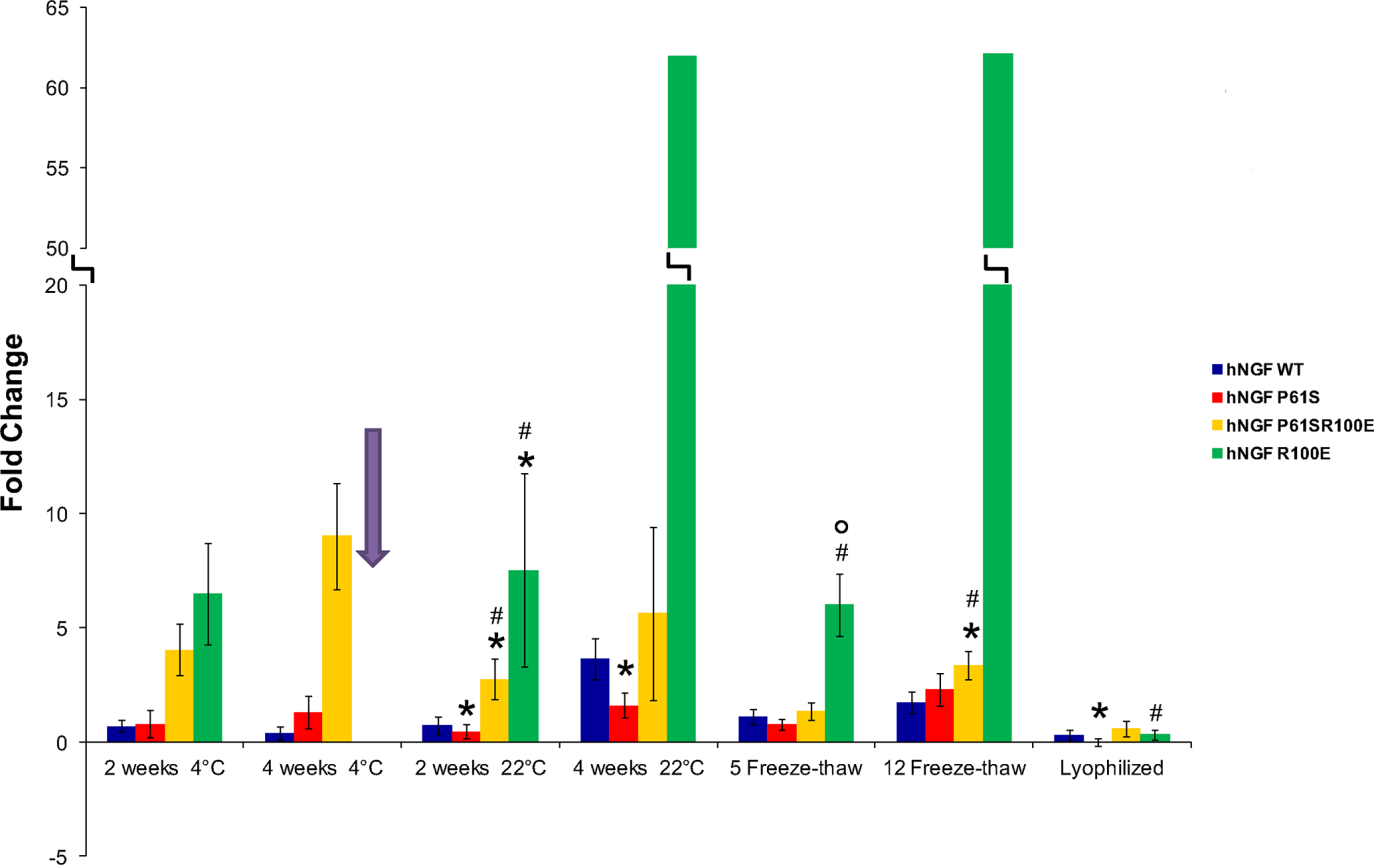
*In vitro* stability by TF1 Proliferation assay read-out. The fold changes of ΔC50 values for all the destabilizing treatments (temperature 4°C-22°C, freeze-thaw cycles 5–12 and lyophilization) carried out on hNGF WT (blue), hNGF P61S (red), hNGF P61SR100E (yellow) and hNGF R100E (green) is shown. The reference curves exhibits a ΔC50 value equal to 0, so that ΔC50 values higher than 0 indicate that the stability of the NGF sample tested was affected. The hNGF R100E fold change in ΔC50 value corresponding to the incubation at 4°C for 2 weeks is missing (arrow). The experimental points corresponding to this treatment could not be interpolated because they did not fit with the theoretical curve. This behavior indicates a strong destabilization of the protein (see S2 Fig). Asterisks represent the samples with a p<0.05 when compared to hNGF WT. Hashes represent the samples with a p<0.05 when compared to hNGF P61S. Circles represent the samples with a p<0.05 when compared to hNGF P61SR100E.

In order to measure the differences in stability between hNGF and mutants after each treatments, a comparison of the ΔC50 values is shown in Fig 7 and in Table 5. The reference curve exhibits a ΔC50 value equal to 0, so that ΔC50 values higher than 0 indicate that the stability of the NGF sample tested was affected.

**Table 5:** A comparison of the ΔC50 values concerning the in vitro stability test of human NGF Wild type and the mutants.

	Reference curve	4°C—2 weeks	4°C—4 weeks	22°C—2 weeks	22°C—4 weeks	5 Freeze-thaw cycles	12 Freeze-thaw cycles	Lyophilization
**hNGF WT**	0.00 ± 0.27	0.70 ± 0.27	0.38 ± 0.29	0.74 ± 0.39	3.66 ± 0.91	1.10 ± 0.33	1.74 ± 0.48	0.30 ± 0.25
**hNGF P61S**	0.00 ± 0.32	0.79 ± 0.58	1.30 ± 0.73	0.46 ± 0.31	1.61 ± 0.55	0.78 ± 0.26	2.32 ± 0.72	0.00 ± 0.17
**hNGF R100E**	0.00 ± 0.19	6.49 ± 2.21	N.A.	7.52 ± 4.22	62.03 ± **437**	6.00 ± 1.36	62.17 ± **194**	0.32 ± 0.21
**hNGF P61SR100E**	0.00 ± 0.21	4.02 ± 1.12	9.02 ± 2.32	2.76 ± 0.88	5.62 ± 3.79	1.36 ± 0.38	3.35± 0.63	0.59 ± 0.34

In order to measure the differences in stability between hNGF and the mutants after the treatments (4°C and 22°C incubation, freeze-thaw cycles, lyophilization) a comparison of the ΔC50 values was evaluated. The reference curve, corresponding to the hNGF or the mutant untreated, exhibits a ΔC50 value equal to 0, so that ΔC50 values higher than 0 indicate that the stability of the NGF sample tested was affected. The ΔC50 for the different treatments were calculated using the formula indicated in the Materials and Methods section. The errors were calculated based on the error propagation formulae. The hNGF R100E mutant exhibits in two treatments high error values (in bold). This is due to the fact that the experimental points corresponding to those treatments did not fit with the theoretical curve, causing high errors. This behavior indicates a strong destabilization of the protein (see S2 Fig).

The hNGF R100E mutant exhibited a high destabilization in all the treatments. This was evident from the experimental points that did not fit with the theoretical dose-response curves, exhibiting high errors (see S2 Fig). In the case of the treatment at 4°C for 4 weeks, the ΔC50 value could not be calculated due to the fact that the data points of the 4 weeks at 4°C incubation could not be interpolated (see S2 Fig). Due to this behaviour, the ΔC50 values corresponding to hNGF R100E for: 4°C at 2 and 4 weeks incubation, 22°C at 4 weeks incubation and 12 freeze-thaw treatment, cannot be treated by the statistical analysis and were thus excluded. Otherwise, when possible, the data were analysed by pairwise Student’s t-test, in all possible combinations, with FDR multiple testing correction.

The ΔC50 data corresponding to 1 week incubation at 4°C and 22°C were not reported in the histogram since the proteins stability did not significantly change with the exception of hNGF R100E. In general, the temperature treatments at 4°C did not affect significantly the stability of all the mutants. hNGF P61SR100E appeared to be slightly destabilized after the incubation at 22°C for 2 weeks, when compared to both hNGF WT and hNGF P61S. hNGF R100E exhibited the highest ΔC50 in almost all the conditions This indicates a significant destabilization of the protein in these conditions, despite the significativity could not be calculated by the statistical test carried out (see S2 Fig)

The freeze-thawing cycles did not affect significantly the stability of the hNGF WT, P61S. hNGF P61SR100E was slightly destabilized after 12 freeze-thawing cycles when compared to hNGF WT and hNGF P61S, while hNGF R100E was highly destabilized in both the treatments. Finally, the lyophilisation treatment did not affect significantly the bioactivity of all the proteins, and it could be used as a procedure to better store all the mutants, in particular hNGF R1000E, mostly affected by all the treatment tested.

In conclusion, hNGF WT and P61S were not destabilized significantly by the treatments, whereas hNGF P61SR100E appeared slightly less stable than hNGF WT and P61S in some of the tested condition. On the other hand, hNGF R100E stability was mostly affected by temperature and freeze and thaw cycles. These data further validate the previously discussed biochemical and biophysical characterization, highlighting the role of structural stability on their bioactivity.

### Binding affinity analysis of NGF mutants: Surface Plasmon Resonance

In order to gain further insights into the impact of the mutations on the properties of NGF, the binding profiles of the mutants against a panel of antibodies, as structural probes, were characterized by Surface Plasmon Resonance. The binding to TrkA and p75^NTR^ receptors have been already investigated [25]. Two different anti-NGF antibodies (mAb 256 R&D System and mAb αD11 [29]), and one anti-proNGF antibody (Millipore, Darmstadt, Germany), were used, in order to verify whether differences in the biophysical and biological behaviour of the various hNGF proteins are reflected also in their interaction kinetic with well established antibody binding partners.

**Fig 8:**
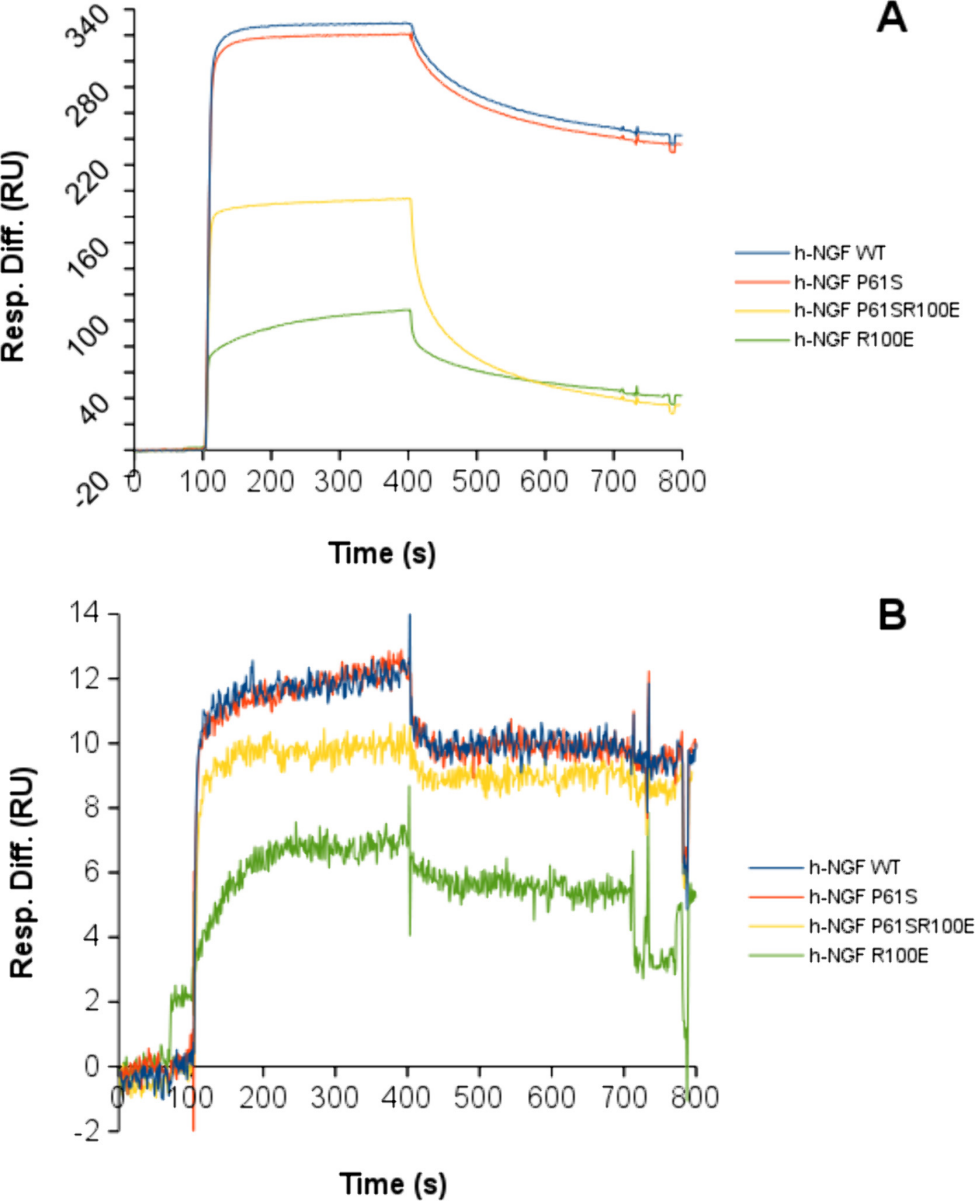
SPR kinetic analysis of NGF and mutants. SPR kinetic analysis of the binding of hNGF WT (blue), hNGF P61S (red), hNGF P61SR100E (yellow), hNGF R100E (green) to the anti-NGF antibodies R&D MAB 256 (panel A) and αD11 (panel B). For each panel, the displayed curves correspond to the higher concentration of the analyte, both for hNGF WT and mutants, in order to compare the neurotrophins on the same antibody. The detailed SPR kinetics are shown in S3–S5 Figs.

From the binding curves (Fig 8), major differences, in the intensities and in the interaction kinetics, were apparent (S3 and S4 Figs, S1 Table), which well compared to those of the hNGFs with both anti-NGF monoclonal antibodies. The binding curves of hNGF WT and hNGF P61S to anti-NGF antibodies were almost overlapping. The hNGF R100E showed a significant impact on its interaction with the anti-NGF antibodies. The hNGF P61SR100E mutant behaved in an intermediate way, indicating that the P61S mutation could somehow mitigate the effects of the R100E mutation. As reported in Tables 6 and 7, the differences in the binding curves reflect differences in the affinity (K_D_ values), the hNGF R100E mutant having a lower affinity than the other mutants.

**Table 6:** Summary of the binding affinities of human NGF wild type and the mutants for the MAb anti-NGF: R&D (MAB 256) and αD11[29] in Surface Plasmon Resonance binding experiments (NGF concentration range: 0.1–100 nM).

	MAb anti-NGF R&D	MAb anti-NGF αD11
**h-NGF WT**	K_D_ = 1.5 nM	K_D_ < pM
**h-NGF P61S**	K_D_ = 2 nM	K_D_ < pM
**h-NGF R100E**	K_D_ = 5 nM	K_D_ = 7 pM
**h-NGF P61SR100E**	K_D_ = 1 nM	K_D_ = 7 pM

**Table 7:** Summary of the binding affinities of human proNGF wild type and the mutants for the MAb anti-NGF: R&D (MAB 256) and αD11 [29] and the MAb anti-proNGF Millipore (clone EP1318Y) in Surface Plasmon Resonance binding experiments (proNGF concentration range: 0.1–100 nM).

	MAb anti-NGF R&D	MAb anti-NGF αD11	MAb anti-proNGF Millipore
**h-proNGF WT**	K_D_ = 30 nM	K_D_ = 4 nM	K_D_ = 5 nM
**h-proNGF P61S**	K_D_ = low	K_D_ = 4 nM	K_D_ = 7 nM
**h-proNGF R100E**	K_D_ = 3 μM	K_D_ = 20 nM	K_D_ = 7 nM
**h-proNGF P61SR100E**	K_D_ = 1 nM	K_D_ = 4 nM	K_D_ = 3 nM

**Fig 9:**
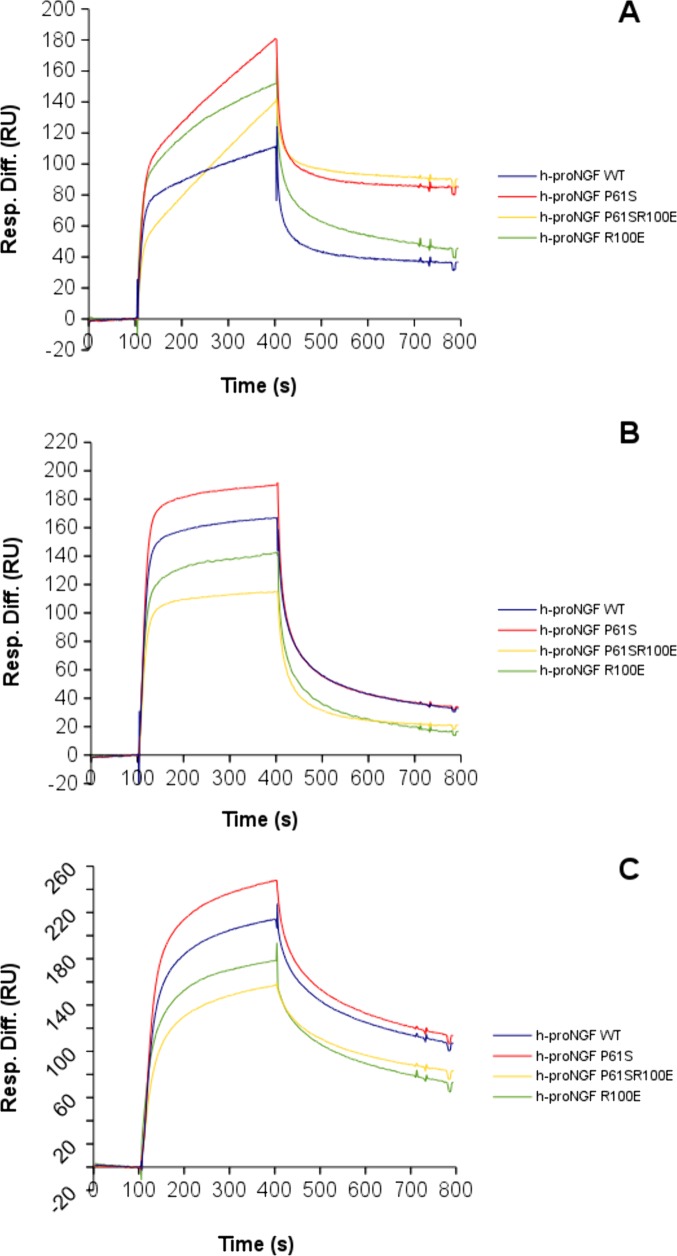
SPR kinetic analysis of NGF and mutants. SPR kinetic analysis of binding of hproNGF WT (blue), hproNGF P61S (red), hproNGF P61SR100E (yellow), hproNGF R100E (green) over the anti-NGF antibodies R&D MAB 256 (panel A), αD11 (panel B), and the anti-proNGF antibodies Millipore clone EP1318Y (panel C). For each panel, the displayed curves correspond to the higher concentration of the analyte, both for hproNGF WT and mutants, in order to compare the proneurotrophins on the same antibody. The detailed SPR kinetics are shown in the S3–S5 Figs.

The hproNGFs mutants interacted with different kinetics and different affinities against anti-NGF and anti-proNGF antibodies, respectively (Table 7, Fig 8, S3–S5 Figs, S1 and S2 Tables). In general, the hproNGFs had lower affinities for the anti-NGF antibodies, than the corresponding hNGFs, as already reported for the interaction between anti-NGF mAb αD11 and mouse proNGF [27, 48]. The proNGFs mutants showed very similar affinities for the anti-proNGF antibody (Table 7), despite some significant differences in their kinetic profiles (see Fig 9 and S5 Fig).

These data provides additional support to the finding that the pro-peptide has an intra-molecular interaction with the mature moiety that in turn influences the interactions of the mature NGF with its specific partners (be it receptors or antibodies). In line with this, the SPR data confirmed an effect of the R100E mutation also on the proNGF domain, even if the mutation is located within the mature NGF moiety. Finally, these data confirmed that the impact of the mutations on hNGFs and hproNGFs is different, depending on the mutation, being greater for the R100E mutation.

### ELISA assay to detect hNGF P61S “tagged” mutants

**Fig 10:**
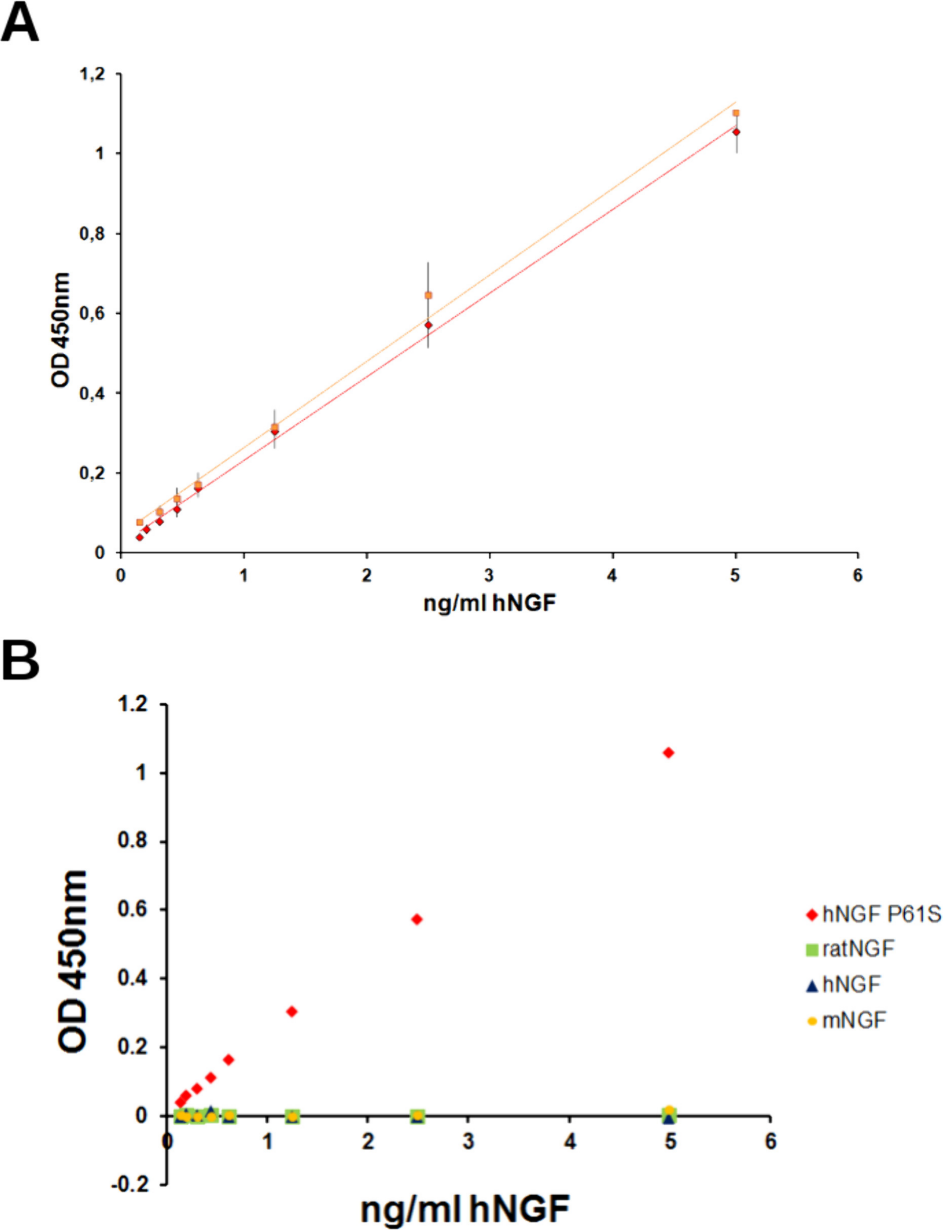
ELISA assay to measure hNGF P61S tagged mutants. Panel A. Standard calibration curve, obtained with hNGF P61S (red diamonds) or hNGF P61SR100E (orange squares). Panel B. Calibration curves carried out with hNGF P61S (red diamonds), rat NGF (green squares), mouse NGF (yellow circles) and hNGF WT (blue triangles). The experimental points are the mean values of the different experiments, the error bars represent the standard deviations.

An immunoassay, able to selectively measure P61S tagged NGF molecules in biological fluids, against the background of endogenous hNGF, was developed. In the optimal format, the anti-NGF 4GA MAb was captured on the plastic wells, the hNGF P61S was used for the standard calibration curve (between 0,15 and 5 ng/ml) and the rabbit anti-NGF polyclonal antibody H20 (Santa Cruz) was used as the detecting antibody. The curve was interpolated linearly and the analytical sensitivity of the assay was established to be 0.15 ng/ml (Fig 10).

In order to confirm the specificity of the assay and the selectivity between hNGF P61S and NGF WT from various species, the calibration curve was carried out using hNGF P61S, as well as mouse, rat and human NGF (Fig 10). The experiment confirmed that hNGF P61S, but not hNGF WT, mouse NGF (mNGF) and rat NGF (rNGF) gives rise to a significant antibody binding. It is important to note that previous studies demonstrated that the anti-NGF MAb 4GA also binds mouse NGF (which has Proline in position 61, like the human P61S mutant) [27] in a direct ELISA format. However in the sandwich assay format presented here, the primary antibody H20 (Santa Cruz) used, specifically binds hNGF. Therefore the antibodies combination used in this sandwich ELISA format, (one, 4GA, specific for the P61S mutation and the second, H-20, specific for human NGF), ensures the specific detection of the P61S tagged molecules versus other forms (hNGF WT or mNGF or rNGF). Thus, therapeutic traceable NGF could be discriminated, against the background of endogenous unmodified NGF in preclinical and clinical studies.

In order to assess the applicability of the ELISA to quantitatively detect the hNGF P61S tagged mutants in biological samples, a set of validation tests were carried out using mouse or rat brain homogenates. Two different concentrations of the recombinant hNGF P61S were spiked into mouse and rat brain tissues, to evaluate the background effect of the biological sample. For each assay, the samples were analyzed in duplicate and the measured values were compared to the theoretical values of the spiking, to obtain the recovery percentage (Table 8). The interference of the biological sample can be considered irrelevant, as shown from the recovery percentages indicated in Table 8, both for mice and rats brain tissues.

**Table 8:** Validation test to evaluate the interference of biological samples on the ELISA assay (mice and rat brain tissues).

Spiking of hNGF P61S tagged (ng/ml)	Mice Brain Tissue		Rat Brain Tissue	
	Recovery % (average of 3 samples)	CV%	Recovery % (average of 22 samples)	CV%
2.5	112	10	156	18
0.625	88	68	88	38

The test (10 assays) was performed by different operators and the results were highly reproducible, (see standard deviation in Fig 10).

The ELISA was carried out also using hNGF P61SR100E for the calibration curve, confirming the same sensitivity and reproducibility.

We conclude that this immunoassay can be exploited to determine the biodistribution of the hNGF mutants containing the traceable tag P61S into mice and rat brain tissue, in preclinical studies.

### Pain sensitivity activity of wild type versus mutant forms of hNGF

**Fig 11:**
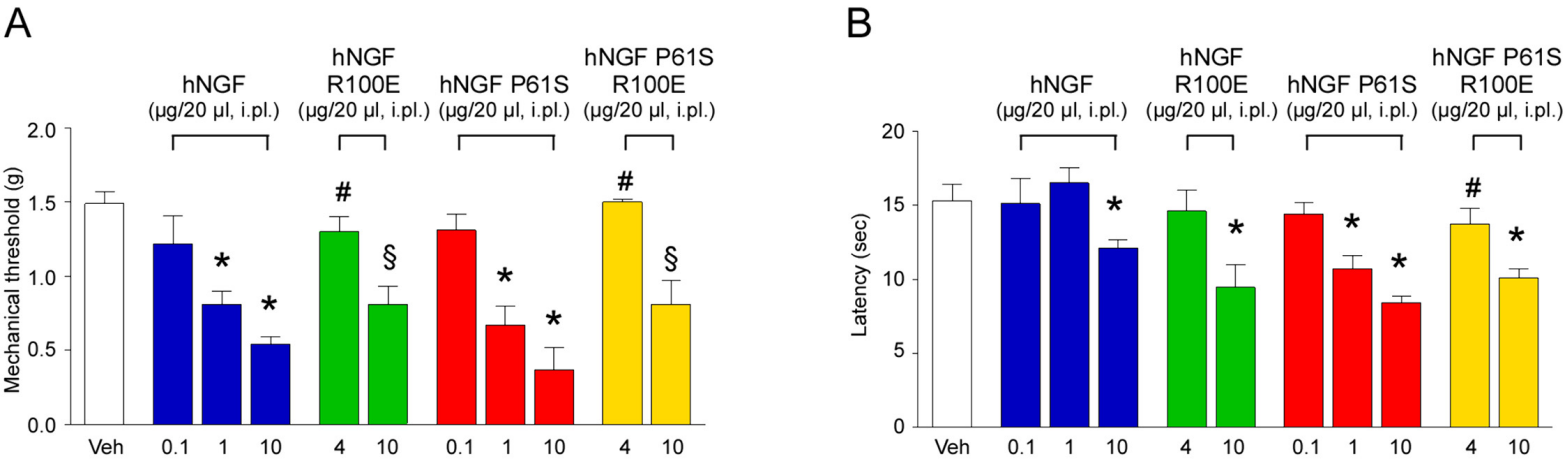
Painful effect induced by hNGF WT or mutants. Pooled data of the mechanical allodynic response (A) and the thermal (hot) hyperalgesic response (B) evoked by intraplantar (i.pl.) injection (20 μl) of hNGF WT, hNGF R100E, hNGF P61S, hNGF P61S R100E or their vehicle (Veh, isotonic saline), measured 5 hours post-treatment. Data are mean ± sem of at least n = 4 mice per group; *P<0.05 vs. Veh or hNGF WT 0.1 μg or hNGF P61S 0.1 μg; #P<0.05 vs. hNGF WT 1 μg or hNGF P61S 0.1 μg; §P<0.05 vs. hNGF WT 10 μg or hNGF P61S 10 μg. One-way ANOVA followed by Bonferroni post-test.

In order to evaluate and compare the pro-nociceptive activity of the various hNGFs, WT and mutants were injected in the hind-paw of adult CD1 mice and the associated mechanical allodynia and heat hyperalgesia were studied. In a previous paper we showed that hNGF WT and the hNGF P61S mutant evoked a time-dependent and dose-dependent mechanical allodynia which was maximal 5 hours after treatments and at the highest dose of 4 μg/20 μl/paw, while the same dose of hNGF R100E and of the double mutant hNGF P61SR100E evoked a reduced allodynia [24]. Here, an additional dose of all mutants have been tested. First, we confirmed that, while hNGF WT and hNGF P61S were already active at the dose of 1 μg/20 μl, the mutants hNGF R100E and hNGF P61SR100E at the dose of 4 μg/20 μl failed to induce pro-nociceptive effects (Fig 11A). Remarkably, while hNGF WT induced mechanical allodynia at 1 μg/20 μl/paw dose (Fig 11 and S6 Fig), the same level of pain was achieved only in response to 10-fold higher concentrations of hNGF R100E or hNGF P61SR100E mutants (10 μg/20 μl/paw).

Controlateral paw showed no significant change in withdrawal thresholds (data not shown). Thus we concluded that both hNGF mutants show a reduced capacity to evoke mechanical allodynia even at a higher dose that what shown before.

Thermal hyperalgesia was assessed by using hot plate assay. In a previous study we showed that the intraplantar (i.pl.) injection of 4 μg/20 μl of hNGF WT induced thermal hyperalgesia from 3 to 5 hours after the injection, while hNGF R100E did not [22]. No data were available for the double mutant hNGF P61SR100E. Here, we measured the hot thermal hyperalgesia evoked by i.pl. injection of wild type or mutant hNGFs, including the double mutant, not only after the injection of 4 μg/20 μl, but analyzing a dose dependent effect during time. hNGF induced thermal hyperalgesia from 3 to 5 hours after the injection of 4 and 10 μg/20 μl, hNGF P61S evoked a dose-dependent thermal hyperalgesia, which was significant already at the dose of 1 μg/20 μl and maximal at 10 μg/20 μl (Fig 11B). The mutant hNGF R100E showed thermal hyperalgesia only at the highest dose used (10 μg/20 μl) (Fig 11B). Similarly, the double mutant hNGF P61SR100E produced a significant pro-nociceptive effect only at the highest dose of 10 μg/20 μl (Fig 11B).

These data underline that hNGF P61SR100E mutant shows a markedly reduced pro-nociceptive activity as compared to hNGF WT and hNGF P61S.

## Discussion

We have previously designed and characterized a hNGF mutant with a reduced nociceptive action, inspired by HSAN V, a rare human genetic disease of congenital insensitivity to pain. This form of hNGF, namely hNGF R100E, shows a reduced nociceptive action, maintaining the neurothrophic activity. We also investigated two more mutants, namely the hNGF P61S, that is selectively detectable against hNGF WT, by using a specific monoclonal antibody, and hNGF P61SR100E, a double mutant, that harbors, beside the painless mutation, the "tagging" mutation that could provide the property of being traceable in biological samples [22–25].

In our previous paper [24], based on the pharmacological efficacy in rescuing phenotype in mice models of neurodegeneration, it was suggested that the mutant hNGF P61SR100E could be considered a therapeutic lead candidate for further development. We now report the structure-activity relationships of the mutants, in both their precursor and mature forms, by putting special emphasis on the properties that a molecule should exhibit in order to be developed as therapeutic candidate by taking a more systematic comparative approach, and studying a number of properties, such as:

- stability as determined by biochemical, biophysical and cellular experimental read-outs;
- structural impact of the mutations based on biophysical measurement both on the mature and the precursor forms
- responsiveness to the TrkA and p75^NTR^ receptors in cellular models
- algesic properties upon injection *in vivo*

Finally, in order to exploit the fundamental traceability property of the P61S mutation, a robust and sensitive immunoassay for the detection of mutants in biological samples was developed.

The stability of the mutants was evaluated, with respect to hNGF WT, by different biochemical/biophysical approaches. Besides the results obtained by thermal stability, in which small differences were observed, it is clear that the P61S mutation does not affect the stability of the protein significantly. On the contrary, the mutation R100E shows a greater destabilization, as seen both in the rate of proteolytic cleavage as well as in the chemical denaturation. The hNGF P61SR100E exhibits an intermediate behavior, highlighting the stabilizing influence of the P61S mutation, which partially mitigates the effect of R100E mutation.

In order to gain further insights in the stability properties of hNGF and mutants, the loss of bioactivity after different treatments has been exploited by using a quantitative biological readout. From the comparison of the ΔC50 value, hNGF WT and P61S appeared not destabilized significantly by the treatments, while hNGF P61SR100E was slightly less stable than hNGF WT and P61S. On the contrary, hNGF R100E stability was greatly affected by all the treatments. These data support the reported biochemical/biophysical studies, and also highlight that a striking difference in their stability has been detected in a cellular context between hNGF R100E and hNGF P61SR100E.

The far-UV circular dichroism results confirmed the differences between the mutants, reflected in different secondary structure composition of the proteins. From the structural point of view, it appears that the P61S mutation shows a very limited influence on the overall structure of mature hNGF, while the R100E mutation has a greater effect, also affecting the secondary structure of the pro-peptide moiety.

**Fig 12:**
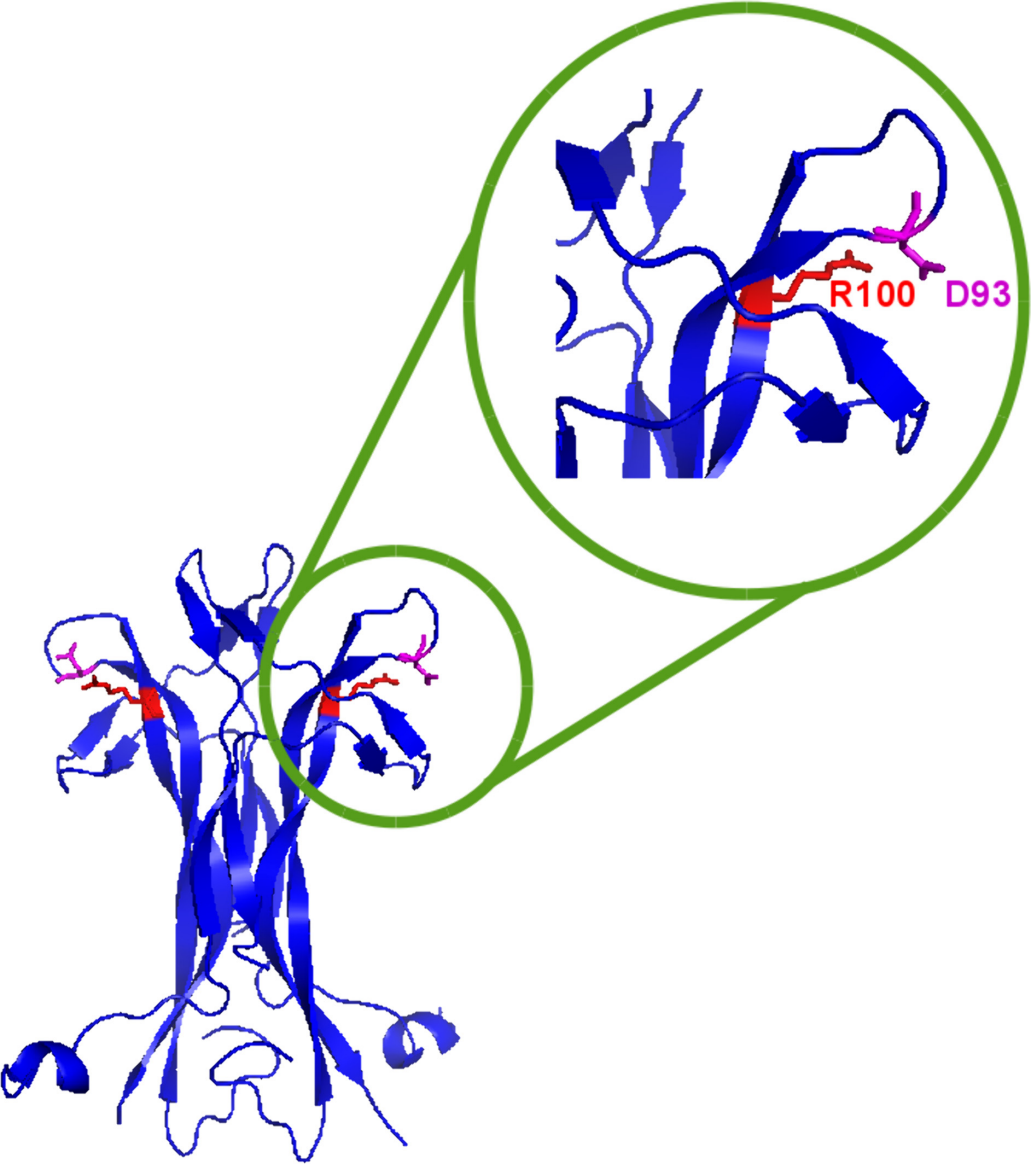
NGF crystallographic structure showing the mutation. NGF crystallographic structure (PDB ID: 1www, in blue) in which the mutated Arginine (R100) and the neighbouring Aspartic Acid (D93), engaged in a ionic bridge, are highlighted in red. Fig 12 has obtained using Pymol [49].

The tridimensional structure of human NGF (PDB (Protein Data Bank): 1www and Fig 12) highlights the residue R100 to be engaged in an electrostatic interaction (salt bridge) with the neighbouring residue D93. The Arginine to Glutamic Acid substitution in the described mutants introduces a local repulsive interaction that may alter the overall loop conformation.

The biophysical characterization also pinpointed differences between the hproNGF R100E and hNGF R100E. Although the mutation resides on the mature protein, the position is close enough to the region of NGF comprising residue W21, that in turn has been suggested to interact with the pro-peptide [27, 42]. Therefore, we reasoned that a change in the loop conformation carrying R100E in the mature protein might influence also the interaction with the pro-peptide. Indeed, our structural studies confirmed that there is a significant interaction between the pro-peptide and the mature domains of murine NGF [46] and that this interaction is probably accounting for the differences observed in the wild-type and mutant proteins, both in their mature and precursor forms. Our structure-activity relationship analysis provides therefore novel insights into the molecular determinants modulating the properties of hNGF mutants.

The receptor specific biological activity of the hNGF mutants was comparatively evaluated by two cellular assays: the proliferation assay on TF1 human erythroleukemic cells, and the differentiation assay of primary rat oligodendrocytes, in order to dissect the mutants functional signalling through the TrkA or the p75^NTR^ receptors, respectively.

The biological activity of the mutants, evaluated using the TF1 assay differs significantly. hNGF WT is the most effective in inducing proliferation in TF1 cells, as expected. hNGF P61S is comparable to hNGF WT in its ability to induce TF1 cells proliferation in a dose/dependent manner, exhibiting a similar C50 value, but a lower Hmax. The painless mutant hNGF R100E shows a decreased Hmax and increased C50 values, even if the C50 is still in the range of low NGF concentrations. hNGF P61SR100E exhibits an intermediate action, as shown by the Hmax and C50 values, falling between those of the hNGF P61S and hNGF R100E mutants.

Moreover, in oligodentrocyte differentiation assay, we found that hNGF WT causes an increase of the percentage of undifferentiated NG2+ cells and a consequent decrease of the percentage of O4+ pre-oligodendrocytes, indicating that hNGF inhibits OPCs differentiation. The effect was absent when OPCs were grown in the presence of hNGFR100E or hNGF P61SR100E. These data show that the single and double R100E mutants are similarly impaired in their ability to engage p75^NTR^, a property of relevance for their receptor signalling profile.

Finally, we extended the studies on the pro-nociceptive effects of the various mutants in mouse. In this experimental setting we measured the ability of both the mutants, hNGF R100E and hNGF P61SR100E, to induce pro-nociceptive effects, mechanical allodynia and thermal hyperalgesia, compared to hNGF and hNGF P61S, respectively. We observed that both hNGF R100E and hNGF P61SR100E were significantly less potent than hNGF WT and hNGF P61S to elicit mechanical allodynia and thermal hyperalgesia, respectively.

Thus, we characterized a set of hNGF mutants with therapeutic potential, aimed at the design of new clinical protocols for the treatment of different kind of diseases, such as Alzheimer's disease, diabetic neuropathies, ophthalmic diseases and dermatological ulcers, where the neurotrophic effects of NGF could be exploited, by avoiding the nociceptive side effects induced by the neurotrophin.

Summing up the results of our previous studies with those presented in this study, we can confirm that, among the different designed mutants, the most promising candidate to advance towards clinical trials is hNGF P61SR100E. Compared with the hNGF R100E, hNGF P61SR100E is characterized by a higher expression level in the production as recombinant protein, by a better ability in induce TF1 proliferation, and by a higher stability.

The observed lower stability of hNGF R100E with respect to hNGF P61R100E rationalize our previous findings described in Capsoni et al. [22, 24], in which we reported that hNGF R100E was less effective than NGF61/100 in rescuing Alzheimer phenotype in AD11 mouse model.

hNGF P61SR100E, moreover, exhibits about 10 fold lower potency in eliciting pain in mouse with respect to hNGF WT and hNGF P61S, a reduction in nociceptive sensitization similar to that shown by hNGF R100E. Present promising *in vivo* data, although obtained in mouse, suggest that the use of the hNGF P61SR100E could increase the therapeutic window for NGF in man. It could be possible, however, that while animal experiments showed less pronociceptive effect, the same might not occur in humans. Further studies with human material may thus be helpful to better understand the nociceptive effects of the hNGF painless, even if the genetic inspiration of these mutants from the pain insensitivity HSANV disease predicts that this protein will also be less painful in humans. In any case, these results provide a strong ground towards performing clinical studies in man. Indeed, the large phase 3 diabetic neuropathy NGF clinical trial failed because the threshold of the administered hNGF WT, that provoked pain in patients, coincided with the minimal pharmacologically effective dose (1 μg/kg) [18]. The hNGF P61SR100E administration could permit to reach the pharmacologically effective dose avoiding nociceptive effect in that therapeutic indication, but, by extension, likely also in other indications as well.

Last but not least, the hNGF P61SR100E mutant, has the additional benefit of being traceable, since the tagging P61S mutation can be recognized by the specific MAb 4GA antibody. To exploit this property, a specific and highly sensitive ELISA assay for the detection of the traceable hNGF P61S mutants in biological samples has been developed. The novel immunoassay could allow high sensitivity measurement of hNGF P61S mutants in biological fluids, in order to track the administered hNGF P61S mutants in preclinical and clinical studies. This feature would then permit to monitor bio-distribution of the drug and precisely define its correct therapeutic window, preventing side effects.

## Supporting Information

S1 Fig Human pre-proNGF aminoacid sequence. The cDNA sequence for human pre-proNGF is reported (UniProt entry P01138).The signal sequence is indicated in italics; the pro-peptide in normal text; mature NGF is indicated in bold. The furin cleavage site is marked as double underline. The sequence of proNGF25 is highlighted by the red box. Position 61 of the mutation P61S is indicated in blue. Position 61 of the mutation R100E is indicated in green.
(DOCX)

S2 Fig hNGF R100E TF1 dose-response curves of *in vitro* stability test. TF1 dose-response curves of the mutant hNGF R100E after incubation at 4°C (2 weeks green triangles, 4 weeks empty circles) and freeze-thaw cycles (5 freeze-thaw orange circles, 12 freeze-thaw red squares) with respect of the untreated control (blue squares). Where possible, the curves were fitted (continuous lines). All the treatments shown in the graph, strongly affected the stability of the mutant hNGF R100E, as evident from the curve shapes. The experimental points referred to the incubation of 4 weeks at 4°C failed to be interpolated with a dose-response curve
(DOCX)

S3 Fig Kinetics of binding of NGF and proNGF mutants over MAb R&D. Detailed SPR binding kinetics of the neurotrophins over the anti-NGF antibody R&D MAb 253. A- h-NGF; B- h-proNGF; C- h-NGF P61S; D- h-proNGF P61S; E- h-NGF R100E; F- h-proNGF R100E; G- h-NGF P61SR100E; H- h-proNGF P61SR100E. Concentrations used, from top to bottom: 100, 50, 25, 6.3, 3.1, 1.6, 0.8, 0.4, 0.2, 0.1 nM.
(DOCX)

S4 Fig Kinetics of binding of NGF and proNGF mutants over MAb αD11. Detailed SPR binding kinetics of the neurotrophins over the anti-NGF antibody MAb αD11. A- h-NGF; B- h-proNGF; C- h-NGF P61S; D- h-proNGF P61S; E- h-NGF R100E; F- h-proNGF R100E; G- h-NGF P61SR100E; H- h-proNGF P61SR100E. Concentrations used, from top to bottom: 100, 50, 25, 6.3, 3.1, 1.6, 0.8, 0.4, 0.2, 0.1 nM.
(DOCX)

S5 Fig Kinetics of binding of NGF and proNGF mutants over MAb Millipore clone EP1318Y. Detailed SPR binding kinetics of the neurotrophins over the anti-NGF antibody MAb Millipore clone EP1318Y. A- h-proNGF; B- h-proNGF P61S; C- h-proNGF R100E; D- h-proNGF P61SR100E. Concentrations used, from top to bottom: 100, 50, 25, 6.3, 3.1, 1.6, 0.8, 0.4, 0.2, 0.1 nM.
(DOCX)

S6 Fig Painful effect induced by hNGF WT and mutants. A) Time- and dose-dependent mechanical allodynic response evoked by intraplantar (i.pl.) injection (20 μl) of hNGF WT, hNGF R100E(left panel) and hNGF P61S, hNGF P61SR100E (right panel) or their vehicle (Veh, isotonic saline). Each point represents the mean ± sem of n≥4 mice; *P<0.05 vs. Veh or hNGF P61S (0.1 μg). One-way ANOVA followed by Bonferroni post-test. B) Time- and dose-dependent thermal (hot) hyperalgesic response induced by i.pl. Injection of hNGF WT, hNGF R100E (left panel) or hNGF P61S and hNGF p61S R100E (right panel) and their Veh. Each point represents the mean ± sem of n≥4 mice; *P<0.05 vs. Veh or hNGF WT (4 μg) or hNGF P61S (1 μg). One-way ANOVA followed by Bonferroni post-test.
(DOCX)

S1 Table Kinetics data of NGF and proNGF WT and mutants. Summary of the kinetic constants of human NGF and proNGF WT and mutant, for the MAb anti-NGF R&D System (MAB 256), the MAb anti NGF αD11 and the MAb anti-proNGF Millipore (clone EP1318Y), extrapolated by the Surface Plasmon Resonance binding experiments.
(DOCX)

S2 Table Comparison between NGF and proNGF K_D_ constant. Summary of the binding affinities of human NGF and proNGF WT and mutants for the MAb anti-NGF: R&D (MAB 256) and αD11 and the MAb anti-proNGF Millipore (clone EP1318Y) in Surface Plasmon Resonance binding experiments.
(DOCX)
