# Supplementary material for: Functional Characterization of Human ProNGF and NGF Mutants: Identification of NGF P61SR100E as a “Painless” Lead Investigational Candidate for Therapeutic Applications
Source: PLoS One. 2015 Sep 15;10(9):e0136425. doi: 10.1371/journal.pone.0136425 (PMC4570711; doi:10.1371/journal.pone.0136425)

**S1 Fig. Human pre-proNGF aminoacid sequence.** The cDNA sequence for human pre-proNGF is reported (UniProt entry P01138).The signal sequence is indicated in italics; the pro-peptide in normal text; mature NGF is indicated in bold. The furin cleavage site is marked as double underline. The sequence of proNGF25 is highlighted by the red box. Position 61 of the mutation P61S is indicated in blue. Position 61 of the mutation R100E is indicated in green.


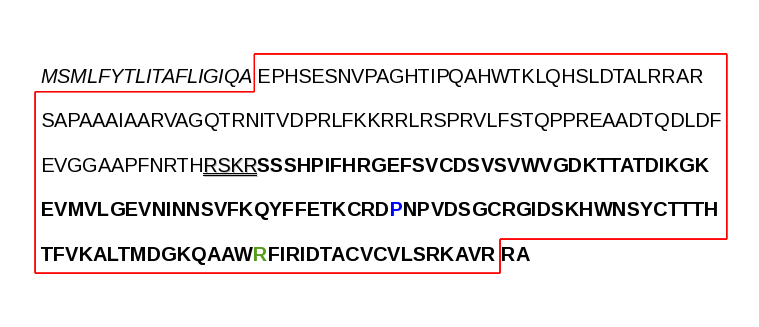

Supplement: S1 Fig — The cDNA sequence for human pre-proNGF is reported (UniProt entry P01138).The signal sequence is indicated in italics; the pro-peptide in normal text; mature NGF is indicated in bold. The furin cleavage site is marked as double underline. The sequence of proNGF25 is highlighted by the red box. Position 61 of the mutation P61S is indicated in blue. Position 61 of the mutation R100E is indicated in green. (DOCX) [file pone.0136425.s001.docx]
