# Supplementary material for: Functional Characterization of Human ProNGF and NGF Mutants: Identification of NGF P61SR100E as a “Painless” Lead Investigational Candidate for Therapeutic Applications
Source: PLoS One. 2015 Sep 15;10(9):e0136425. doi: 10.1371/journal.pone.0136425 (PMC4570711; doi:10.1371/journal.pone.0136425)
Supplement: S2 Fig — TF1 dose-response curves of the mutant hNGF R100E after incubation at 4°C (2 weeks green triangles, 4 weeks empty circles) and freeze-thaw cycles (5 freeze-thaw orange circles, 12 freeze-thaw red squares) with respect of the untreated control (blue squares). Where possible, the curves were fitted (continuous lines). All the treatments shown in the graph, strongly affected the stability of the mutant hNGF R100E, as evident from the curve shapes. The experimental points referred to the incubation of 4 weeks at 4°C failed to be interpolated with a dose-response curve (DOCX) [file pone.0136425.s002.docx]

**S2 Fig. hNGF R100E TF1 dose-response curves of *in vitro* stability test.** TF1 dose-response curves of the mutant hNGF R100E after incubation at 4°C (2 weeks green triangles, 4 weeks empty circles) and freeze-thaw cycles (5 freeze-thaw orange circles, 12 freeze-thaw red squares) with respect of the untreated control (blue squares). Where possible, the curves were fitted (continuous lines). All the treatments shown in the graph, strongly affected the stability of the mutant hNGF R100E, as evident from the curve shapes. The experimental points referred to the incubation of 4 weeks at 4°C failed to be interpolated with a dose-response curve
