# Supplementary material for: Functional Characterization of Human ProNGF and NGF Mutants: Identification of NGF P61SR100E as a “Painless” Lead Investigational Candidate for Therapeutic Applications
Source: PLoS One. 2015 Sep 15;10(9):e0136425. doi: 10.1371/journal.pone.0136425 (PMC4570711; doi:10.1371/journal.pone.0136425)

**S3 Fig. Kinetics of binding of NGF and proNGF mutants over MAb R&D.** Detailed SPR binding kinetics of the neurotrophins over the anti-NGF antibody R&D MAb 253. A- h-NGF; B- h-proNGF; C- h-NGF P61S; D- h-proNGF P61S; E- h-NGF R100E; F- h-proNGF R100E; G- h-NGF P61SR100E; H- h-proNGF P61SR100E. Concentrations used, from top to bottom: 100, 50, 25, 6.3, 3.1, 1.6, 0.8, 0.4, 0.2, 0.1 nM.


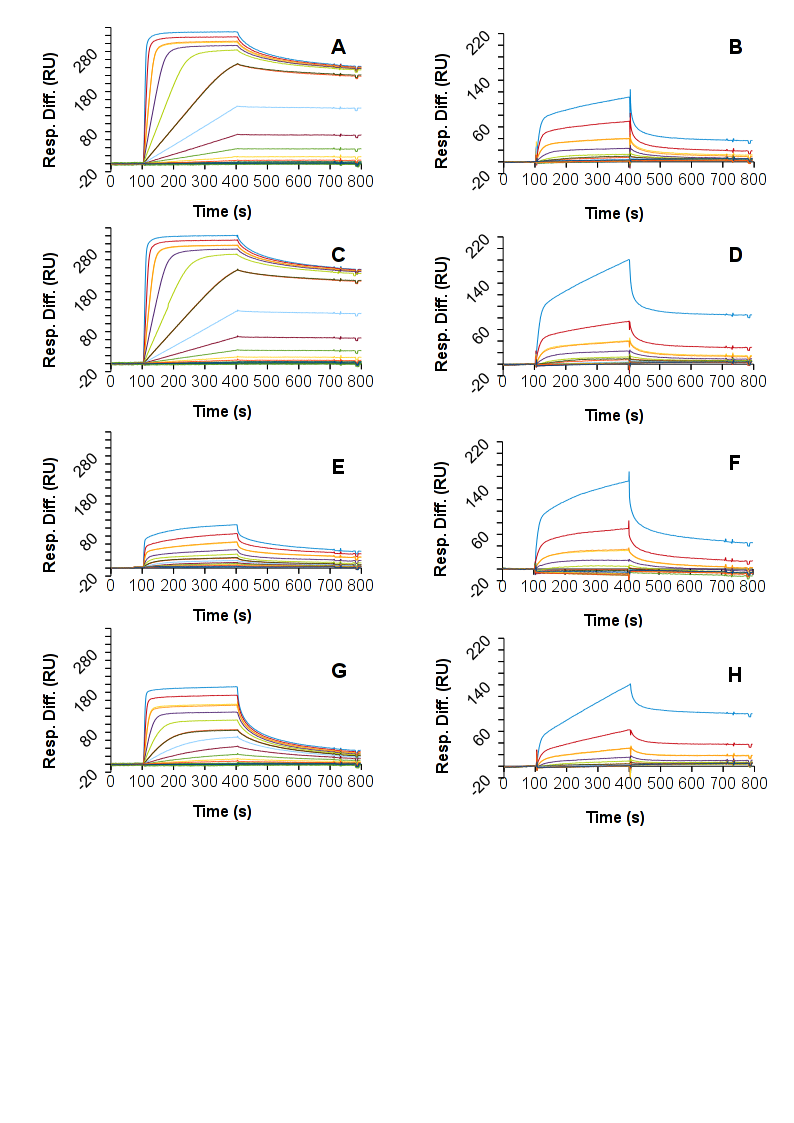

Supplement: S3 Fig — Detailed SPR binding kinetics of the neurotrophins over the anti-NGF antibody R&D MAb 253. A- h-NGF; B- h-proNGF; C- h-NGF P61S; D- h-proNGF P61S; E- h-NGF R100E; F- h-proNGF R100E; G- h-NGF P61SR100E; H- h-proNGF P61SR100E. Concentrations used, from top to bottom: 100, 50, 25, 6.3, 3.1, 1.6, 0.8, 0.4, 0.2, 0.1 nM. (DOCX) [file pone.0136425.s003.docx]
