# Supplementary material for: Functional Characterization of Human ProNGF and NGF Mutants: Identification of NGF P61SR100E as a “Painless” Lead Investigational Candidate for Therapeutic Applications
Source: PLoS One. 2015 Sep 15;10(9):e0136425. doi: 10.1371/journal.pone.0136425 (PMC4570711; doi:10.1371/journal.pone.0136425)

**S6 Fig. Painful effect induced by hNGF WT and mutants.** A) Time- and dose-dependent mechanical allodynic response evoked by intraplantar (i.pl.) injection (20 µl) of hNGF WT, hNGF R100E(left panel) and hNGF P61S, hNGF P61SR100E (right panel) or their vehicle (Veh, isotonic saline). Each point represents the mean ± sem of n≥4 mice; *P<0.05 vs. Veh or hNGF P61S (0.1 µg). One-way ANOVA followed by Bonferroni post-test. B) Time- and dose-dependent thermal (hot) hyperalgesic response induced by i.pl. Injection of hNGF WT, hNGF R100E (left panel) or hNGF P61S and hNGF p61S R100E (right panel) and their Veh. Each point represents the mean ± sem of n≥4 mice; *P<0.05 vs. Veh or hNGF WT (4 µg) or hNGF P61S (1 µg). One-way ANOVA followed by Bonferroni post-test.


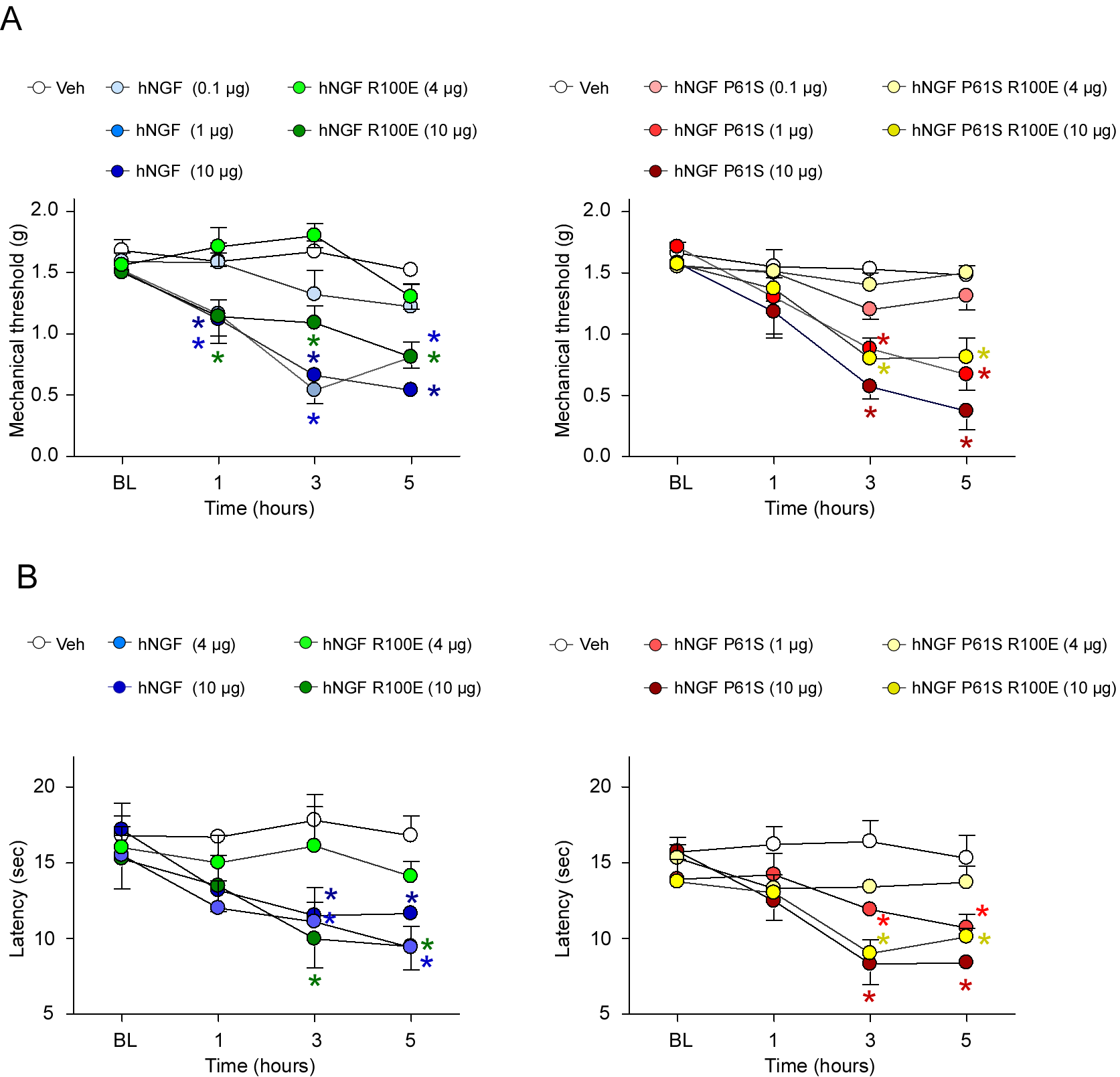

Supplement: S6 Fig — A) Time- and dose-dependent mechanical allodynic response evoked by intraplantar (i.pl.) injection (20 μl) of hNGF WT, hNGF R100E(left panel) and hNGF P61S, hNGF P61SR100E (right panel) or their vehicle (Veh, isotonic saline). Each point represents the mean ± sem of n≥4 mice; *P<0.05 vs. Veh or hNGF P61S (0.1 μg). One-way ANOVA followed by Bonferroni post-test. B) Time- and dose-dependent thermal (hot) hyperalgesic response induced by i.pl. Injection of hNGF WT, hNGF R100E (left panel) or hNGF P61S and hNGF p61S R100E (right panel) and their Veh. Each point represents the mean ± sem of n≥4 mice; *P<0.05 vs. Veh or hNGF WT (4 μg) or hNGF P61S (1 μg). One-way ANOVA followed by Bonferroni post-test. (DOCX) [file pone.0136425.s006.docx]
