# Supplementary material for: Functional Characterization of Human ProNGF and NGF Mutants: Identification of NGF P61SR100E as a “Painless” Lead Investigational Candidate for Therapeutic Applications
Source: PLoS One. 2015 Sep 15;10(9):e0136425. doi: 10.1371/journal.pone.0136425 (PMC4570711; doi:10.1371/journal.pone.0136425)
Supplement: S1 Table — Summary of the kinetic constants of human NGF and proNGF WT and mutant, for the MAb anti-NGF R&D System (MAB 256), the MAb anti NGF αD11 and the MAb anti-proNGF Millipore (clone EP1318Y), extrapolated by the Surface Plasmon Resonance binding experiments. (DOCX) [file pone.0136425.s007.docx]

**S1 Table. Kinetics data of NGF and proNGF WT and mutants.** Summary of the kinetic constants of human NGF and proNGF WT and mutant, for the MAb anti-NGF R&D System (MAB 256), the MAb anti NGF αD11 and the MAb anti-proNGF Millipore (clone EP1318Y), extrapolated by the Surface Plasmon Resonance binding experiments.

|  | **hNGF WT** | **hNGF P61S** | **hNGF R100E** | **hNGF P61SR100E** |
| --- | --- | --- | --- | --- |
| **MAb anti-NGF R&D** | k_a_ =8.3e6 1/M s  k_d_ =0.9e-3 1/s  χ^2^=7 | k_a_ =7.9e6 1/M s  k_d_ =0.9e-3 1/s  χ^2^=5 | k_a_ =0.23e6 1/M s  k_d_ =1.3e-3 1/s  χ^2^=15 | k_a_ =6.6e6 1/M s  k_d_ =8.5e-3 1/s  χ^2^=8 |
| **MAb anti-NGF αD11** | k_a_ =4.3e6 1/M s  k_d_ =3.6e-7 1/s  χ^2^=0 | k_a_ =4.4e6 1/M s  k_d_ =0.3e-7 1/s  χ^2^=0 | k_a_ =16e6 1/M s  k_d_ =1.2e-4 1/s  χ^2^=0 | k_a_ =3.5e6 1/M s  k_d_ =0.3e-4 1/s  χ^2^=0 |
|  | **hproNGF WT** | **hproNGF P61S** | **hproNGF R100E** | **hproNGF P61SR100E** |
| **MAb anti-NGF R&D** | k_a_ =5.4e4 1/M s  k_d_ =1.9e-3 1/s  χ^2^=6 | k_a_ =0.4e4 1/M s  k_d_ =1.0e-3 1/s  χ^2^=13 | k_a_ =0.1e4 1/M s  k_d_ =2.5e-31/s  χ^2^=16 | k_a_ =7.5e4 1/M s  k_d_ =646 1/s  χ^2^=7 |
| **MAb anti-NGF αD11** | k_a_ =1.2e6 1/M s  k_d_ =4.9e-3 1/s  χ^2^=8 | k_a_ =1.1e6 1/M s  k_d_ =5.2e-3 1/s  χ^2^=9 | k_a_ =0.3e6 1/M s  k_d_ =5.1e-3 1/s  χ^2^=3 | k_a_ =1.3e6 1/M s  k_d_ =5.9e-3 1/s  χ^2^=7 |
| **MAb anti-proNGF Millipore** | k_a_ =2.3e5 1/M s  k_d_ =1.2e-3 1/s  χ^2^=18 | k_a_ =1.8e5 1/M s  k_d_ =1.3e-3 1/s  χ^2^=27 | k_a_ =2.1e5 1/M s  k_d_ =1.6e-3 1/s  χ^2^=32 | k_a_ =3.5e5 1/M s  k_d_ =1.2e-3 1/s  χ^2^=27 |
