# Supplementary material for: Functional Characterization of Human ProNGF and NGF Mutants: Identification of NGF P61SR100E as a “Painless” Lead Investigational Candidate for Therapeutic Applications
Source: PLoS One. 2015 Sep 15;10(9):e0136425. doi: 10.1371/journal.pone.0136425 (PMC4570711; doi:10.1371/journal.pone.0136425)
Supplement: S2 Table — Summary of the binding affinities of human NGF and proNGF WT and mutants for the MAb anti-NGF: R&D (MAB 256) and αD11 and the MAb anti-proNGF Millipore (clone EP1318Y) in Surface Plasmon Resonance binding experiments. (DOCX) [file pone.0136425.s008.docx]

**S2 Table. Comparison between NGF and proNGF K_D_ constant.** Summary of the binding affinities of human NGF and proNGF WT and mutants for the MAb anti-NGF: R&D (MAB 256) and αD11 and the MAb anti-proNGF Millipore (clone EP1318Y) in Surface Plasmon Resonance binding experiments.

|  | **MAb anti-NGF R&D** | **MAb anti-NGF αD11** | **MAb anti- proNGF Millipore** |
| --- | --- | --- | --- |
| **hNGF WT** | 1.5 nM | < pM | 5 μM |
| **hproNGF WT** | 30 nM | 4 nM | 5 nM |
| **hNGF P61S** | 2 nM | < pM | 6 μM |
| **hproNGF P61S** | low | 4 nM | 7 nM |
| **hNGF P61SR100E** | 1 nM | 7 pM | 1 μM |
| **hproNGF P61SR100E** | 1 nM | 4 nM | 3 nM |
| **hNGF R100E** | 5 nM | 7 pM | 1 nM |
| **hproNGF R100E** | 3 nM | 20 nM | 7 nM |
